# Study of Machining Process of SiCp/Al Particle Reinforced Metal Matrix Composite Using Finite Element Analysis and Experimental Verification

**DOI:** 10.3390/ma13235524

**Published:** 2020-12-03

**Authors:** Rashid Ali Laghari, Jianguang Li, Yongxiang Wu

**Affiliations:** Harbin Institute of Technology, School of Mechatronics Engineering, Harbin 150001, China; rashidali@stu.hit.edu.cn (R.A.L.); wu_yx@hit.edu.cn (Y.W.)

**Keywords:** SiCp/Al metal matrix composite, finite element analysis, constitutive model, cutting force prediction, surface morphology, chip morphology, stress distribution, machining process

## Abstract

In this paper, a two-dimensional orthogonal cutting simulation model of SiCp/Al composite was established. The geometry and material constitutive model of the particle, the matrix, and the interface layer have been modeled respectively. In view of the distribution of the particles in the matrix, this paper proposed respectively a two-dimensional particle random distribution to simulate particles randomly distributed in the matrix. Then, the cutting state of SiC reinforced particles was analyzed, the novel approach was adopted the geometric shapes of SiC particles in this study is taken as an oval shape. Three different locations of SiC particle relative to the cutting tool path were simulated to analyze the cutting state such as particle removal. The interface layer was introduced to the case that the particle was on the cutting path to study the influence on the stress and strain transfer. Through the post-processing of simulation results, the influence of interface property on the composite reinforcement effect was studied quantificationally. Finally, the cutting process of SiCp/Al composite material was simulated. This paper studied the influence on the machined surface morphology, chip morphology, stress distribution, and cutting force of many factors of the cutting speed and the cutting thickness. The single factor orthogonal cutting experiment was designed the influence of cutting speed and feed rate on the cutting force. The cutting force results of the experiment and the simulation were compared through the deviation analysis, which verified the simulation model.

## 1. Introduction

In cutting simulation research, two simulation models are commonly used. One is an equivalent homogeneous model. In the simulation, the size, shape, and position distribution of particles in SiCp/Al metal matrix composite material is a kind of material, which uses aluminum alloy as the matrix phase and SiC particles as the reinforcing phase. It has good wear resistance, good dimensional stability, corrosion resistance, low thermal expansion coefficient, good thermal conductivity, high specific strength, and other excellent material properties [1,2]. SiCp/Al composite possesses robust applications in several different areas. Some applications of SiCp/Al includes optical precision instruments, aerospace industries, medical device manufacturing, modern landing gears, electronic packaging, helicopter blades, sports equipment, and automotive parts manufacturing [3,4]. At the same time, the presence of high-hardness SiC particles makes SiCp/Al composites a difficult-to-machine material [5,6]. In the cutting process, the tool wear is severe, and the defects on the machined surface are more. These factors seriously hinder the several application of the SiCp/Al composite material [7,8]. 

In cutting simulation research, two simulation models are commonly used. One is an equivalent homogeneous model. In the simulation, the size, shape, and position distribution of particles in the material are neglected and described by the material constitutive equation. The mechanical behavior of the composite material as a whole [9,10,11]; another model is the multiphase mixing model. Considering the morphology and distribution of the particles, the material properties of the particles and the matrix are defined respectively, which can simulate the interaction between the particles, etc. [12,13]. The partial failure behavior can also simulate the overall mechanical behavior in the composite cutting process [14,15]. 

The SiC particle-enhanced phase acts as a strengthening effect on the matrix, but at the same time, it increases the difficulty for machining, making the machining performance of the SiCp/Al composite material different from the general metal material [16,17]. Machining is currently the most widely-used cutting method with different cutting positions and situations such as the model developed by Ramesh et al. [18] which is based on four cutting situations: Cutting tool contact with SiC particles, cutting tool cut through SiC particle, cutting tool contact aluminum matrix, and cutting tool cut through aluminum matrix, and they studied SiCp/Al composite chip formation process and the distribution of normal and shear stresses in the workpiece during cutting. The results show that the maximum stress in the chip contact area is at a certain distance from the tool tip, and it is pointed out that the distribution position of SiC particles plays an important role in the stress distribution of the workpiece.

In order to study the material’s ultimate shear stress at the tool–chip interface during the titanium alloy machining process the influence of friction coefficient on the ultimate shear stress at the tool–chip interface was taken into consideration, and an associated friction coefficient was proposed by Mabrouki et al. [19]. A model of ultimate shear stress was proposed. The final result of simulation was compared with the cutting experimental data in related kinds of literatures and validates the accuracy of the models. Fathipour et al. [20] used ABAQUS/Explicit software version 6.14-1, company located in Johnston, Newark, NJ, USA to establish a two-dimensional microscopic model of SiCp/Al composites materials. In this model, particles and matrix materials are separately modeled. The SiC particles with 20 volume fraction were taken, which were treated as an elastic material. Binding constraints were imposed after assembling the interface between particles matrix interface tied together. The Johnson–Cook constitutive model was used in this study. Cutting force and chip formation morphology were investigated. The fluctuations in cutting force were observed by authors. The validity of the established finite element model was verified by comparing the chip morphology of the simulation prediction and experimental observation under different feed rates. The analysis was performed on the machining process with simulation methods and mechanisms considered by several researchers, which has reflected many aspects and provides a rich understanding of the machining process. The research mechanisms adopted by Shui et al. [21] give a thorough understanding of the geometry of the SiC particles in the simulation. The author considered the study behavior of different geometric shapes of particles, the parametric modeling of the different shapes and volume fraction of particles by the script language Python on the finite element ABAQUS software were studied. The author established three finite element geometric models with particle shapes: Regular polygon blend, arbitrary polygon, and circular particles; through comparison, it was found that during the cutting process, the shape of the particles has an important effect on the size of the stress distribution. The cutting forces of the round and regular polygon shapes are stable and the stress is small. Furthermore, through analysis of the machining process simulation of different mechanical models the research mechanisms well-thought-out by Ghandehariun et al. [22] proposed the micromechanical FE model of machining MMCs, it proposes the behavior of all components that distinguish MMC such as particles, matrix, and the particle–matrix interface. Fracture, de-bonding, and other scenarios of tool particle interactions were studies through this model. Predicted forces generated by the model were compared with the obtained experimental data and verified. In addition to mechanical modeling of machining process of SiCp/Al composites, Pramanik et al. [23] proposed the FE cutting simulation model, divided the position distribution between the particles and the cutting path into three cases: Particles distributed above the cutting path, particles along the cutting path, and particles below the cutting path. The analysis of the interaction between the cutting tool and SiC particles during orthogonal cutting of SiCp/Al6061 composite materials with 20% SiC particles were used for study. The results show that the sliding of the particles on the contact surface of the tool is the main cause of tool wear. The particle distribution of the stress at the unused position of the tool will fluctuate critically. Finite element simulation performance is increasingly demanding for machining while considering different aspects, e.g., Zhu et al. [24] proposed the interface failure model which is based on the interface failure between the particles and the matrix. Using the ABAQUS/Explicit finite element software to simulate 10%-Al_2_O_3_/Al6061 composite material with an orthogonal machining process was taken to study. The results demonstrate that the comparison of the particles in another deformation zones, the equivalent positive stress on the particles in the first deformation zone is larger. The shear stress on the particles is negative in the first deformation zone turns to a positive value in the second deformation zone. Gallab et al. [25,26] conducted a high-speed dry milling machining operations under different cutting parameters when studying the machining performance of SiC/Al 20wt.% composite materials. The study emphasized the comprehensive tool wear model, surface quality, and the degree of damage under the surface. The strength of polycrystalline diamond PCD tools it meets the requirements and finds that high-speed milling reduces the surface quality.

In this paper, a two-dimensional orthogonal cutting simulation model for establishing SiCp/Al composites is developed based on the finite element method. The constitutive models of the matrix, particles, and interface layers will be constructed separately. On this basis, the random distribution of particles will be simulated and the particles will be studied through simulation. FE Simulated the influence on the machined surface morphology, chip morphology, stress distribution, and cutting force with different factors such as cutting speed and the cutting thickness and different volume fraction SiC particles. The single factor orthogonal cutting experiment was designed the influence of cutting speed and feed rate on the cutting force while keeping the depth of cut constant. The simulated cutting processes of SiCp/Al composite materials verifying the simulated cutting forces by right-angle cutting experiments.

## 2. SiCp/Al Metal Matrix Composite Cutting Simulation Modeling Procedures

### 2.1. The Construction of the Matrix Constitutive Model

The Johnson–Cook constitutive model has been widely used in metal cutting simulation research because of its good anti-conversion and high efficiency. The Johnson–Cook model considers the influence of strain, strain rate, temperature, etc. The Johnson–Cook damage law for simulation was adopted by several researchers due to its easy use and a good description of the material in FEM [27,28] on the flow stress and can be expressed as:

(1) $$\sigma =\left[A+B\varepsilon^{n}\right]\left[1+c\;ln\left(\frac{\overset{•}{\varepsilon }}{{\overset{•}{\varepsilon }}_{0}}\right)\right]\left[1-{\left(\frac{T-T_{r}}{T_{m}-T_{r}}\right)}^{m}\right]$$

where *T_r_* and *T_m_* represent room temperature (K) and material melting point (K) respectively, the equivalent plastic strain rate and reference strain rate; m is the material thermal softening coefficient; *A* represents the yield strength of the material at room temperature; and *n*, *c* respectively represent material strain and strain rate enhancement factors.

Aluminum matrix material parameters and Johnson–Cook constitutive model parameters are shown in Table 1 and Table 2, respectively.

### 2.2. Construction of Particle Constitutive Model

SiC material is a typical brittle-hard material, and the brittle fracture failure of SiC particles easily occurs in the cutting process of SiCp/Al composites. In the FEM cutting simulation, it is necessary to consider the behavior of the SiC particles after the start of fracture and the failure of the fracture. Therefore, the Brittle Cracking Material Model was introduced in this study as reported in [29]. The SiC particles were simulated using elliptical particles. The SiC particle material parameters are shown in Table 3.

The SiC particles are in a state of elasticity before fracture, and the stress–strain relationship obeys Hooke’s law. The judgment criterion of the fracture initiation adopts the maximum normal stress criterion.

(2) $$max(\sigma_{1},\sigma_{2},\sigma_{3})=\sigma_{0}$$

In the equation, σ_1_, σ_2_, σ_3_ is the principal stress component (MPa); σ_0_ is the material tensile stress (MPa). The failure evolution of SiC particles after fracture failure is controlled by the fracture energy criterion. Failure crack initiation displacement is defined as:

(3) $$max(\sigma_{1},\sigma_{2},\sigma_{3})=\sigma_{0}$$

(4) $$u_{n0}=2G_{f}^{I}/\sigma_{tu}^{I}.$$

In the equation, $G_{f}^{I}$ it is the type I fracture energy (J/m^2^); $\sigma_{tu}^{I}$ it is the failure stress (MPa) at the time of crack formation. In order to describe the effect of shear stress on the evolution of particle failure, a functional shear stress retention model for the crack initiation strain is introduced. The shear modulus of SiC particulates during failure evolution *G*_c_ is calculated as follows:

(5) $$G_{c}=\rho \left(e_{nn}^{ck}\right)G.$$

In the equation *G* is the shear modulus before material failure; $e_{nn}^{ck}$ is the shear transfer coefficient.

(6) $$\rho \left(e_{nn}^{ck}\right)=(1-\frac{e_{nn}^{ck}}{e_{max}^{ck}{}^{p}}).$$

In the equation, *p*,$\;e_{max}^{ck}$ is the material related parameters [30], in this study, the value of 1 and 0.001; $e_{nn}^{ck}$ is the cracking strain.

The relevant parameters in the brittle cracking material model based on references and calculations are shown in Table 4.

### 2.3. Interface Cohesion Model Construction

**Figure 1:**
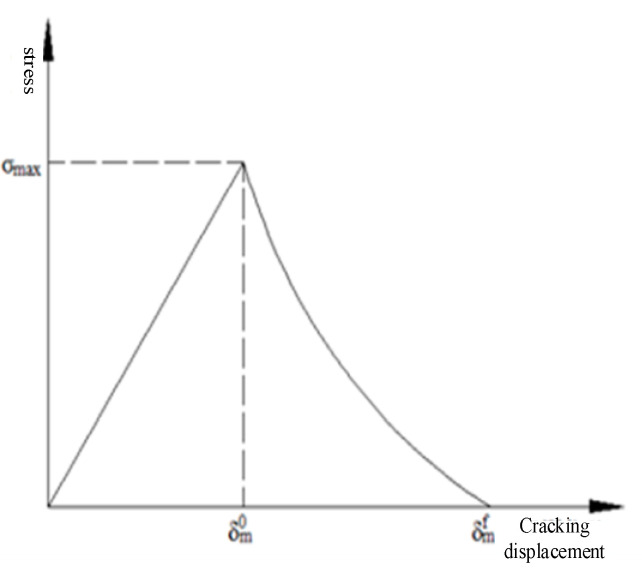
Tension relationship of exponential model.

In this study, the modeling of the interface layer uses a cohesive force model based on cohesive elements. The simulation of cohesive zone elements for utilization in FE models is provided by [31]. This type of element was also adopted by Tvergaard [32] in his study for simulating de-bonding phenomena of the fiber matrix interface whisker reinforced aluminum metal matrix composite. The failure process of the cohesion force model specifically shows that the stress increases as the cracking displacement increases and the damage begins when it increases to the highest point; then the stress appears to decrease and the component enters the damage expansion phase; when the stress value ends When it is reduced to zero, the component completely destroys the failure [33]. The concrete constitutive model of the interface layer adopts an exponential model. As shown in Figure 1, the tension and displacement are linear before the material reaches the strength limit, and the tension and displacement are exponential after the material reaches the strength limit.

The maximum nominal stress criterion is used to determine the initial damage start:

(7) $$max\left\{\frac{<\sigma_{n}>}{\sigma_{n}^{0}},\frac{\sigma_{s}}{\sigma_{s}^{0}}\right\}=1$$

In the equation, σ*_n_*, σ*_s_* the normal and tangential components (MPa) $\sigma_{n}^{0},\;\sigma_{s}^{0}$ of the stress are the maximum nominal normal stress (MPa) and the tangential stress (MPa), respectively. Once the initial damage criterion is reached, the stiffness of the cohesive element starts to decay according to the damage coefficient D. The relationship between the stress and the damage coefficient D is shown in Equation (8).

(8) $$\begin{array}{cc} \sigma_{n}= & \left\{\begin{array}{cc} \left(1-D\right)\bar{\sigma_{n}}, & \sigma_{n}\geq 0\\ \bar{\sigma_{n}}, & \bar{\sigma_{n}}<0 \end{array}\right.\\ & \sigma_{s}=\left(1-D\right)\bar{\sigma_{s}} \end{array}$$

In equation $\bar{\sigma_{n}}$, $\bar{\sigma_{s}}$ they are the stress components when they are not damaged.

Damage coefficient *D* can be calculated by Equation (9).

(9) $$D=1-\left\{\left\{\frac{\delta_{m}^{0}}{\delta_{m}^{max}}\right\}\left\{1-\frac{1-exp\left[-\alpha \left(\frac{\delta_{m}^{max_{m}^{0}}}{\delta_{m}^{f}-\delta_{m}^{0}}\right)\right]}{1-exp\left(-\alpha \right)}\right\}\right\}$$

In the equation $\delta_{m}^{max}$ is the maximum effective displacement in the loading process; $\delta_{m}^{0}$ the initial effective displacement of the damage; $\delta_{m}^{f}$ the interface completely invalid effective displacement. The cohesive force model was compiled using the ABAQUS custom material subroutine VUMAT. The input of the custom material subroutine VUMAT mainly includes the material parameter matrix and the state variable storage matrix. The specific parameter settings of the material parameter matrix are shown in Table 5. Where, $\sigma_{n}^{max}$, $\sigma_{s}^{max}$ is the maximum normal and tangential stress, *δ_n_*, *δ_s_* and is the cracking displacement corresponding to the maximum normal stress and the maximum tangential stress; *T*_0_ is the initial thickness of the element; *E* is the elastic modulus, which characterizes the stiffness of the interface layer.

The specific parameter settings of the state variable matrix are shown in Table 6. Where *ε*_22_, *ε*_11_, *ε*_33_, and *ε*_12_ are the updated values of the strain variable *Δ_n_*, *Δ_s_* the cracking displacements of the normal and tangential directions of the element, $\Phi_{n}^{0},\;\Phi_{s}^{0}$ the updated values of the normal and tangential critical fracture energy, respectively, and Fra is the mark variable of the element deletion.

In the VUMAT subroutine of the cohesion model, firstly, the displacement of the interface is calculated from the increment of the strain, and then the stress of the interface cracking process is calculated through the tension displacement relationship.

### 2.4. Construction of SiCp/Al Composite Constitutive Model

After establishing the constitutive model of the three-phase material of matrix, particle, and interface layer, in order to describe the overall mechanical behavior of the material in the SiCp/Al composite cutting simulation model, the distribution of particles in the matrix needs to be simulated.

The shapes of silicon carbide particles in SiCp/Al composites are different in this study. At the present stage number of research studies were conducted on the round particles, which are commonly used to simulate silicon carbide particles, given that oval particles are closer to the shape of real silicon carbide particles than round particles (such as the long diameter of an ellipse). The ratio of the ratio and the ellipse is different in the two-dimensional area. For the sake of simulation efficiency, the elliptic particles are used to simulate silicon carbide particles.

To illustrate the implementation of the algorithm, first give a specific situation in which the elliptical particles are randomly distributed within a rectangular region: The composite material is simulated with a rectangle in a two-dimensional region and its size is (1) {−4, 4} × {3, 3}; elliptic particles are randomly distributed in a rectangular region, the length of the long axis a is randomly distributed in the interval (2) [0.04, 0.08], and the length of the short axis b satisfies (3):

(10) $$b=\left(0.6+t\right)\cdot a$$

where *t* is in the interval (4) [0, 0.04] are randomly distributed, and the angle *θ* between the long axis and the plane x-axis is randomly distributed in the *θ* interval [0, π], and the particles account for 30% of the total material.

The basic idea of the algorithm is as follows: Elliptical particles are randomly distributed in a rectangular matrix, but particles cannot make contact with each other. For this reason, a recognition area is set around each generated elliptical particle so that other particles cannot enter. If a newly generated ellipse does not exist, Contact with the previous ellipse (contained or intersected by the previous ellipse) and the ellipse is contained within the rectangular region, the ellipse is accepted, otherwise the ellipse is discarded and the new ellipse is regenerated; when the ellipse satisfying the condition reaches the desired. When the area ratio (i.e., the particle volume fraction of the SiCp/Al composite) is reached, the algorithm ends and the final result is output.

**Figure 2:**
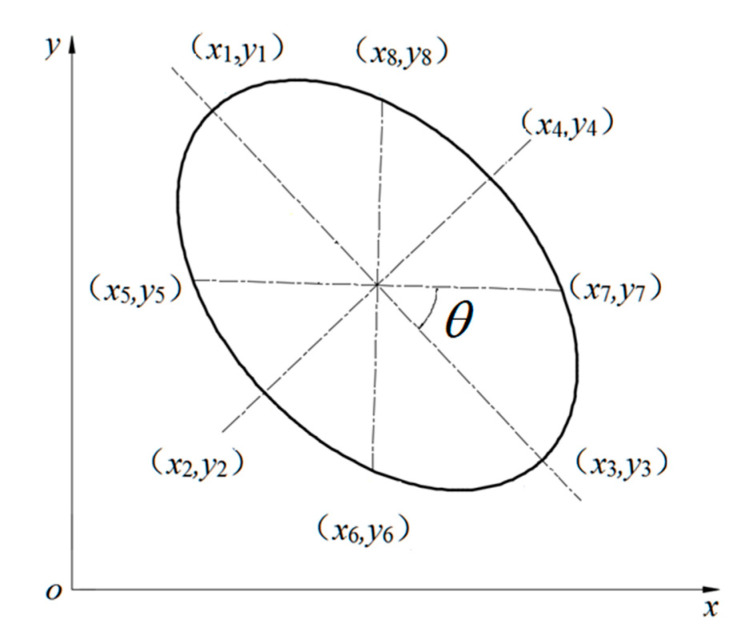
Schematic diagram of ellipse reference point.

The following describes the geometric basis and various criteria for the algorithm:

1. The coordinates of the center point are (*x*_0_, *y*_0_), the length of the major axis is *a*, the length of the minor axis is *b*, and the ellipse equation at any position where the angle between the major axis and the *x*-axis of the plane is *θ* as shown in Equation (11).
  (11) $$\frac{1}{a^{2}}{\left[\left(x-x_{0}\right)\cdot cos\theta +\left(y-y_{0}\right)\cdot sin\theta \right]}^{2}+\frac{1}{b^{2}}{\left[\left(y-y_{0}\right)\cdot cos\theta -\left(x-x_{0}\right)\cdot sin\theta \right]}^{2}=1$$
2. Cover the ellipse that was generated with a slightly larger ellipse (long and short axes are respectively 1.2 times of the original ellipse) as the recognition area, which is called covering ellipse. To reduce the amount of calculation, select eight special positions of the ellipse ((*x_i_*, *y_i_*), *i* = 1–8) as a reference point, as shown in Figure 2. If the reference point of the newly generated ellipse is not included by the cover ellipse of the previous ellipse, it is determined that the overlay condition is satisfied. From the coordinate transformation equation, the coordinates of each reference point are calculated as shown in Equations (13)–(20):
  (12) $$c=\sqrt{\frac{a^{2}b^{2}}{a^{2}+b^{2}}}$$
  (13) $$\left\{\begin{array}{c} x_{1}=b\cdot sin\theta +x_{0}\\ y_{1}=b\cdot cos\theta +y_{0} \end{array}\right.$$
  (14) $$\left\{\begin{array}{c} \;x_{2}=-a\cdot cos\theta +x_{0}\\ y_{2}=a\cdot sin\theta +y_{0} \end{array}\right.$$
  (15) $$\left\{\begin{array}{c} x_{3}=-b\cdot sin\theta +x_{0}\\ y_{3}=-b\cdot cos\theta +y_{0} \end{array}\right.$$
  (16) $$\left\{\begin{array}{c} x_{4}=a\cdot cos\theta +x_{0}\\ y_{4}=-a\cdot sin\theta +y_{0} \end{array}\right.$$
  (17) $$\left\{\begin{array}{c} x_{5}=-c\cdot cos\theta +c\cdot sin\theta +x_{0}\\ y_{5}=c\cdot sin\theta +c\cdot cos\theta +y_{0} \end{array}\right.$$
  (18) $$\left\{\begin{array}{c} x_{6}=-c\cdot cos\theta -c\cdot sin\theta +x_{0}\\ y_{6}=c\cdot sin\theta -c\cdot cos\theta +y_{0} \end{array}\right.$$
  (19) $$\left\{\begin{array}{c} x_{7}=c\cdot cos\theta -c\cdot sin\theta +x_{0}\\ y_{7}=-c\cdot sin\theta -c\cdot cos\theta +y_{0} \end{array}\right.$$
  (20) $$\left\{\begin{array}{c} x_{8}=c\cdot cos\theta +c\cdot sin\theta +x_{0}\\ y_{8}=-c\cdot sin\theta +c\cdot cos\theta +y_{0} \end{array}\right.$$
3. Coverage Condition: The reference point of the newly generated ellipse does not come into contact with the cover ellipse of the previous ellipse. Substituting reference point coordinates into functions $F\left(x,y\right)$, this $F\left(x_{i},y_{i}\right)\geq 0$. When it is determined that the newly generated ellipse satisfies the coverage condition, $\left(x_{i},y_{i}\right)$ where is the reference point, *i* = 1–8 $F\left(x,y\right).$ An expression of the function is given in Equation (21).
  (21) $$F\left(x,y\right)=\frac{1}{a^{2}}{\left[\left(x-x_{0}\right)\cdot cos\theta +\left(y-y_{0}\right)\cdot sin\theta \right]}^{2}+\frac{1}{b^{2}}{\left[\left(y-y_{0}\right)\cdot cos\theta -\left(x-x_{0}\right)\cdot sin\theta \right]}^{2}-1$$
4. Boundary conditions: The generated ellipse does not intersect with the rectangular boundary, i.e., the ellipse is contained within the rectangle (Ecuation (22)).
  (22) $$\left\{\begin{array}{c} -4\leq x_{i}\leq 4\\ -3\leq y_{i}\leq 3\; \end{array}\right.,i=0,1,\ldots ,8$$
5. Area condition: The sum of the area of the ellipse satisfying the coverage condition and the area condition is 30% of the area of the rectangle, and the equation of the area of the ellipse is shown in Equation (23).
  (23) S=π⋅a⋅b

**Figure 3:**
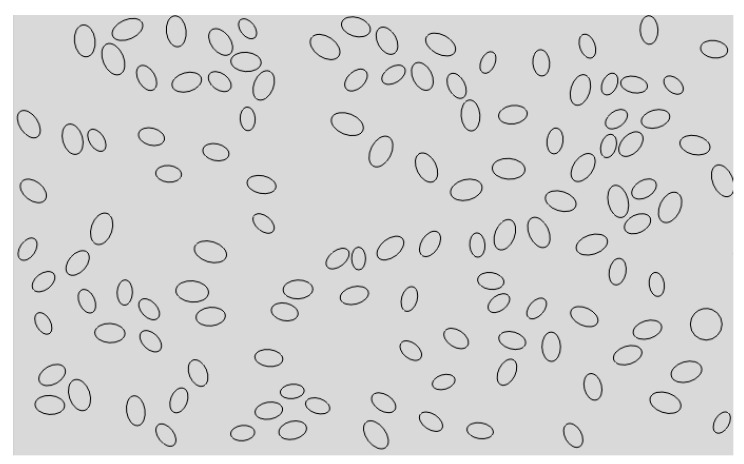
2D sketch of the model.

**Figure 4:**
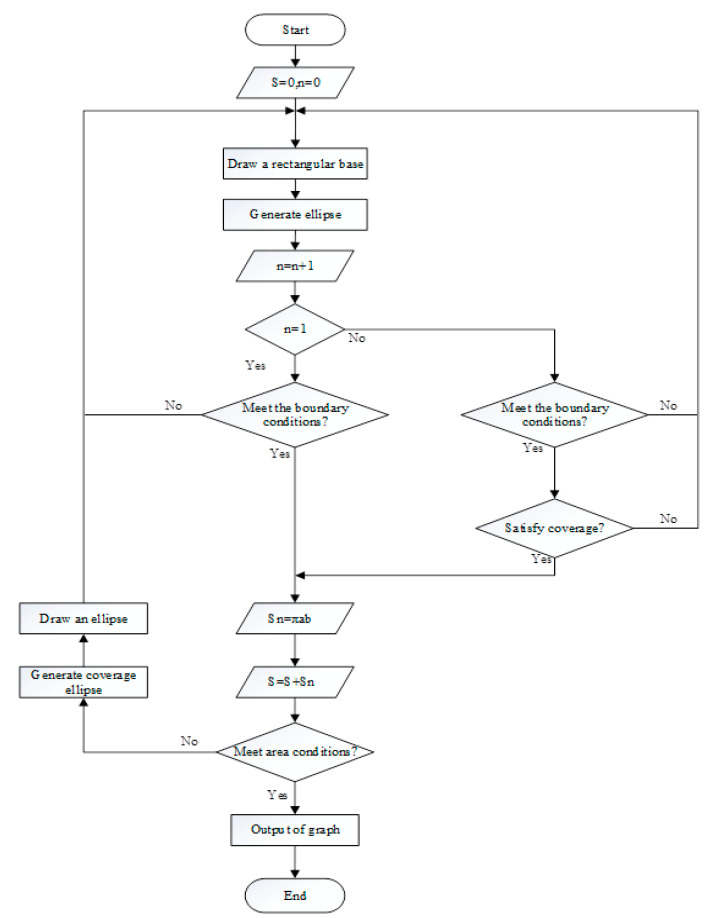
Flow chart of particle random distribution algorithm.

**Figure 5:**
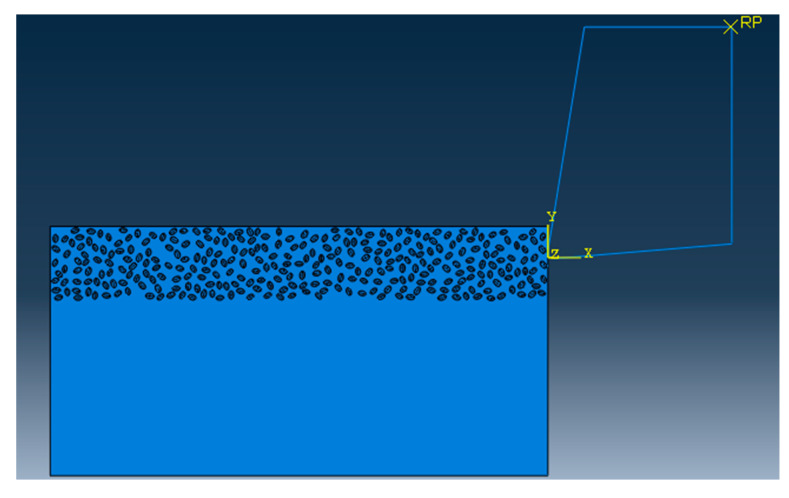
Two-dimensional orthogonal cutting finite element simulation models.

Using Python language to implement the algorithm, and scripting the secondary development of ABAQUS, so that the particles of different sizes and different volume fractions, which can be randomly distributed in the matrix. The final two-dimensional sketch of the ABAQUS finite element model is shown in Figure 3. It can be seen that the simulation results have achieved the desired results. The flow chart of the random particle distribution algorithm is shown in Figure 4. After the random distribution of the particles in the matrix is completed, the components are assembled together by binding constraints. The constitutive relations of the matrix, particles, and interface layers are described by their respective constitutive models. The transfer of stresses and displacements between three-phase materials is achieved by binding constraints, thus describing the overall constitutive relationship of SiCp/Al composites. The final two-dimensional orthogonal cutting simulation model is shown in Figure 5.

### 2.5. Meshing and Boundary Conditions

A two-dimensional orthogonal cutting simulation model of SiCp/Al 50% volume fraction was studied. The Material constitutive model along with the geometry of the matrix, particle, and interface layer was constructed to study the metal cutting process. In the metal cutting simulation process meshing of the component or cutting tool plays a vital role in the final result of the simulated model. In this simulation modeling of the cutting tool was adopted as two-dimensional analytical rigid bodies to simplify the model reasonably and reduce computing costs. When meshing, both the SiC particles and the Al matrix adopt the free mesh method, and the unit uses the CPE4RT unit.

CPE4RT type of the mesh was adopted in this model, the particles were structured using structured mesh, and the accuracy of the simulated model was related to the choice of mesh and its density. The interfacial layer components were developed with the cohesive element, which was separately bound with matrix and particle with tie constraints for transmitting the displacement and cutting force so that the contact surface of the particles and the substrates had a sufficient contact stiffness. The boundary conditions were fixed at the nodes at the bottom and left edges of the model. Cutting forces achieved during simulated models with different mesh seeds were used for the analysis of mesh sensitivity.

**Figure 6:**
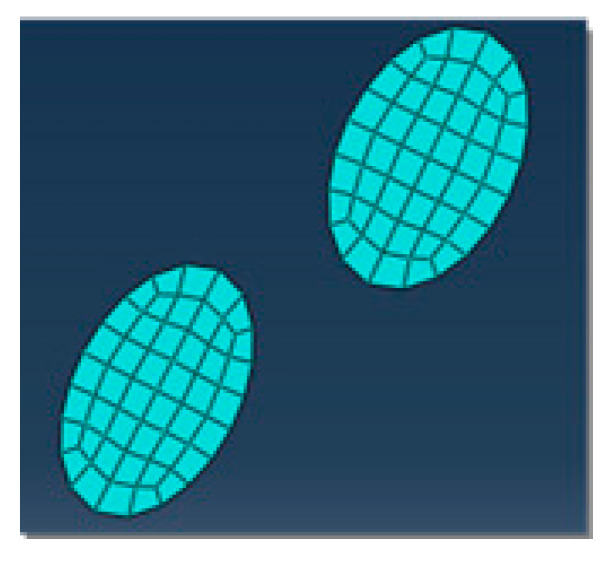
Particle meshing.

**Figure 7:**
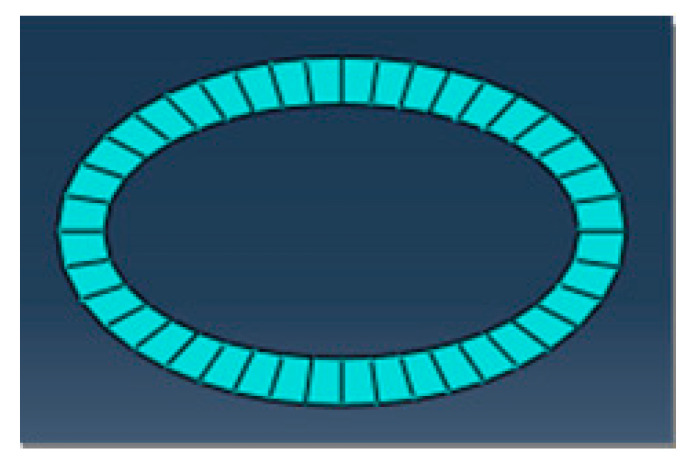
Interface layer meshing.

**Figure 8:**
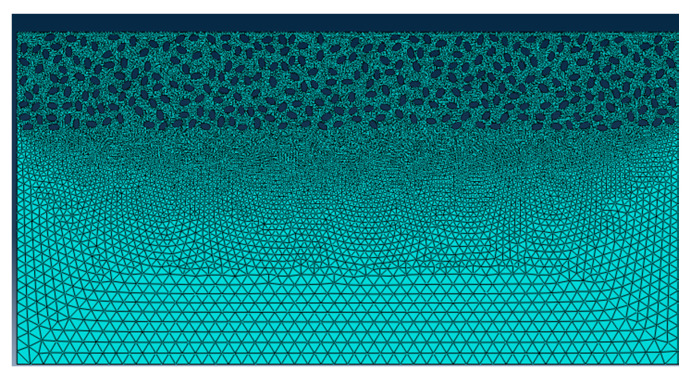
Matrix meshing.

**Figure 9:**
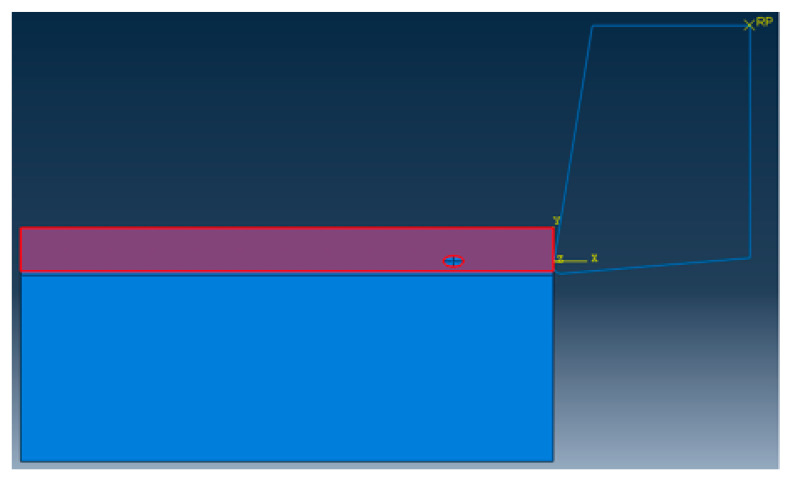
Contact setting of tool and cutting layer.

**Figure 10:**
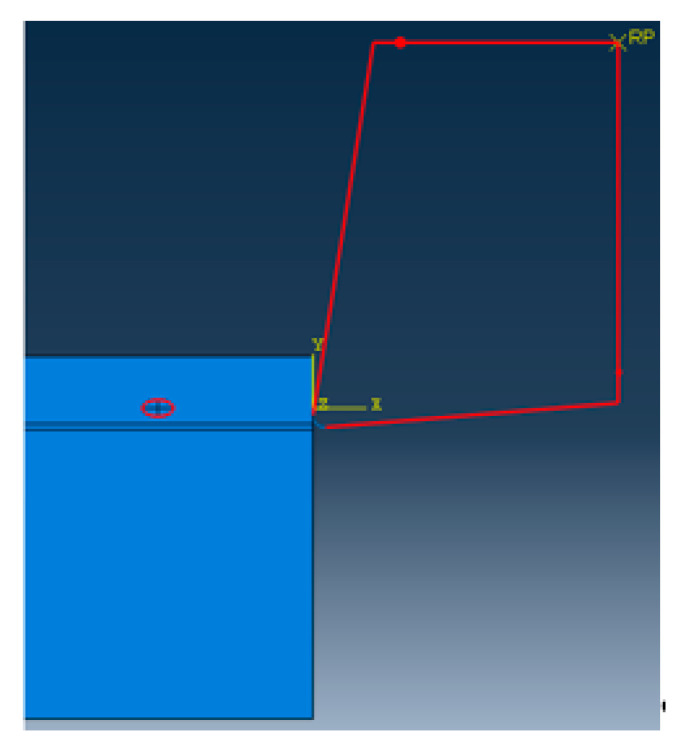
Contact setting of tool and particle.

The meshing of the matrix was also divided into free meshes. The mesh type for the matrix in this model was used as a CPE3T. The matrix meshing, particle meshing, and interface layer meshing are shown in Figure 6, Figure 7 and Figure 8. Particles below the workpiece cutting layer have little effect on the simulation results; to improve the calculation efficiency, only the particles near the cutting layer were retained. The contact settings include the contact pattern of the tool with the cutting layer and the contact between the tool and the particles, as shown in Figure 9 and Figure 10.

Finally, to simulate the SiC particles and the interface between the substrates, the particles and the substrate were bound together using the Tie property so that the contact surface of the particles and the substrate had sufficient contact stiffness. De-bonding was achieved while the failure of the matrix occurred with is tied with particles (these phenomena were also used by [20,23,34]).

### 2.6. Selection of Chip Separation Criteria

In order to achieve material failure and then some of the material separation and formation of cuttings, must be a reasonable choice of chip separation criteria to be consider in machining process. A reasonable cutting model can describe the mechanical properties of the material during cutting deformation and can obtain reasonable prediction results such as cutting profile and cutting force. Liu et al. [35] studied the influence of several commonly-used fracture criteria in finite element simulation modeling on the machining process. The results show that the Johnson–Cook fracture model, considering the evolution of damage, can describe the forming mechanism of the chip and predict the actual stress, strain, etc. Therefore, the Johnson–Cook fracture model considering the evolution of damage was selected in this study.

In the Johnson–Cook fracture model, which includes the evolutionary stages of failure, the material begins to fail at the highest point of the material’s stress–strain curve, and then the material’s stiffness slowly decreases to the point where it completely fails.

- Material failure initiation: When the failure parameter *ω* = 1, the material starts to fail and is determined by Equation (24):
  (24) $$\omega =\sum \frac{∆{\bar{\varepsilon }}^{\mathrm{pl}}}{{\bar{\varepsilon_{0}}}^{\mathrm{pl}}}$$
  where $∆{\bar{\varepsilon }}^{\mathrm{pl}}$—equivalent strain increases of material,$\;{\bar{\varepsilon_{0}}}^{\mathrm{pl}}$—The equivalent strain at failure can be obtained from Equation (25):
  (25) $${\bar{\varepsilon_{0}}}^{\mathrm{pl}}=\left[d_{1}+d_{2}exp(d_{3}\frac{p}{q})\right]\left[1+d_{4}\ln(\frac{\overset{•}{\bar{\varepsilon }}}{\overset{•}{\bar{\varepsilon_{0}}}})\right]\left[1+d_{5}\left(\frac{T-T_{room}}{T_{melt}-T_{room}}\right)\right]$$
  where $d_{1}$~$d_{5}$— material failure model coefficient.
  p/q—p means principal stress, *q* flow stress.

From the above formula, the equivalent stress of material failure is determined by the stress ratio, equivalent strain and temperature.

- Evolving stage of material failure after the failure begins; the damage evolution criterion can be applied to numerical simulation based on equivalent plastic displacement ${\bar{u}}^{\mathrm{pl}}$ or fracture energy $G_{f}$ determination. The fracture energy required to form a unit area crack can be expressed as:
  (26) $$G_{f}=\int_{{\bar{\varepsilon_{0}}}^{\mathrm{pl}}}^{{\bar{\varepsilon_{f}}}^{\mathrm{pl}}}L_{c}\bar{\sigma }d{\bar{\varepsilon }}^{\mathrm{pl}}=\int_{0}^{{\bar{u_{f}}}^{\mathrm{pl}}}\bar{\sigma }d{\bar{u}}^{\mathrm{pl}}$$
  where—${\bar{u}}^{\mathrm{pl}}$ the equivalent displacement in the evolution phase, —$L_{c}$ unit feature length.

Based on the above analysis, reference parameters and Johnson–Cook failure model parameters for calculating the available matrix materials are shown in Table 7.

### 2.7. Turning Machining Experiment Design

**Figure 11:**
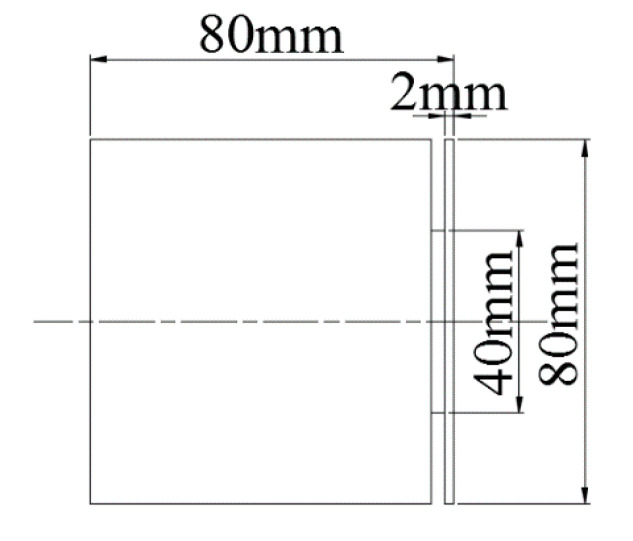
Workpiece diagram.

**Figure 12:**
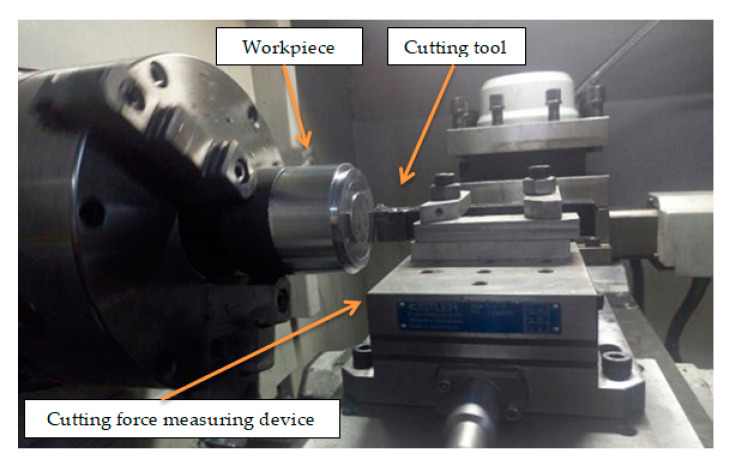
Actual turning diagram.

In this turning experiment, SiCp/Al 50%vol composites were turned using a right-angle cutting method with an average particle size of 10μm. The experimental machine tool used CAK50135di CNC lathes produced with a spindle power of 6.5 kW, and its maximum spindle speed can reach 1450 r/min, the fast forward speed of the machine tool is 1900 mm/min by Shenyang Machine Tool Plant (Shenyang, China). The tool uses a PCD turning tool with a rake angle of 0°, a relief angle of 6°, and a tool inclination of 0°. In order to realize right-angle free cutting, a cylindrical workpiece with a diameter of 80 mm and a height of 80 mm is machined as shown in Figure 11. The actual turning process is shown in Figure 12.

In this experiment, a single-factor experimental order was used to study the influence of different cutting parameters on the cutting force, and the simulation model was verified by comparing the experimental cutting force results with the simulated cutting force results. There are two variables, namely cutting speed and feed rate depth of cut remain constant. There is only one-factor variation in each group of experiments and all other factors remain unchanged. Specific experimental parameters are shown in Table 8.

**Figure 13:**
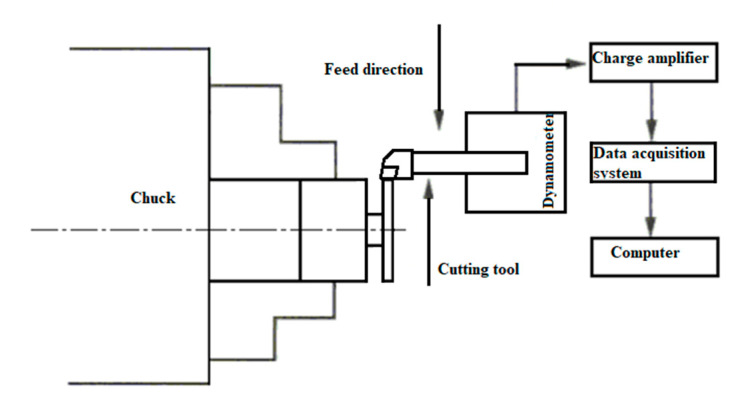
Cutting force measurement system.

The cutting force measurement system is shown in Figure 13. Among them, the tool is clamped on the dynamometer, and the dynamometer is fixedly connected with the worktable. The cutting force measurement system consists of a Kistler three-way dynamometer, a charge amplifier, a data acquisition card, and a computer equipped with data analysis software DynoWare software.

## 3. Result and Discussion

**Figure 14:**
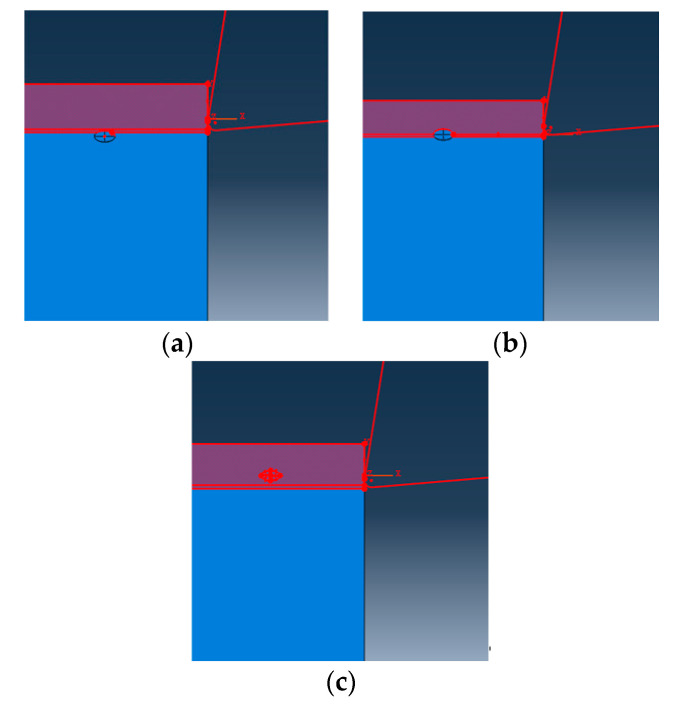
Three different positions of particles, (**a**) particle below the line, (**b**) particle on the line, and (**c**) particle above the line.

SiC particles are the main factors affecting the performance of SiCp/Al composites. It is necessary to study the cutting and strengthening properties of SiC particles. In the model, only one SiC particle is set. Three typical scenarios are studied for the relative positional relationship between the particle and the tool path. The particle is located on the tool cutting path, the particle is located above the tool path, and the particle is located below the tool path, as shown in Figure 14.

### 3.1. Particles That Are on the Cutting Path

**Figure 15:**
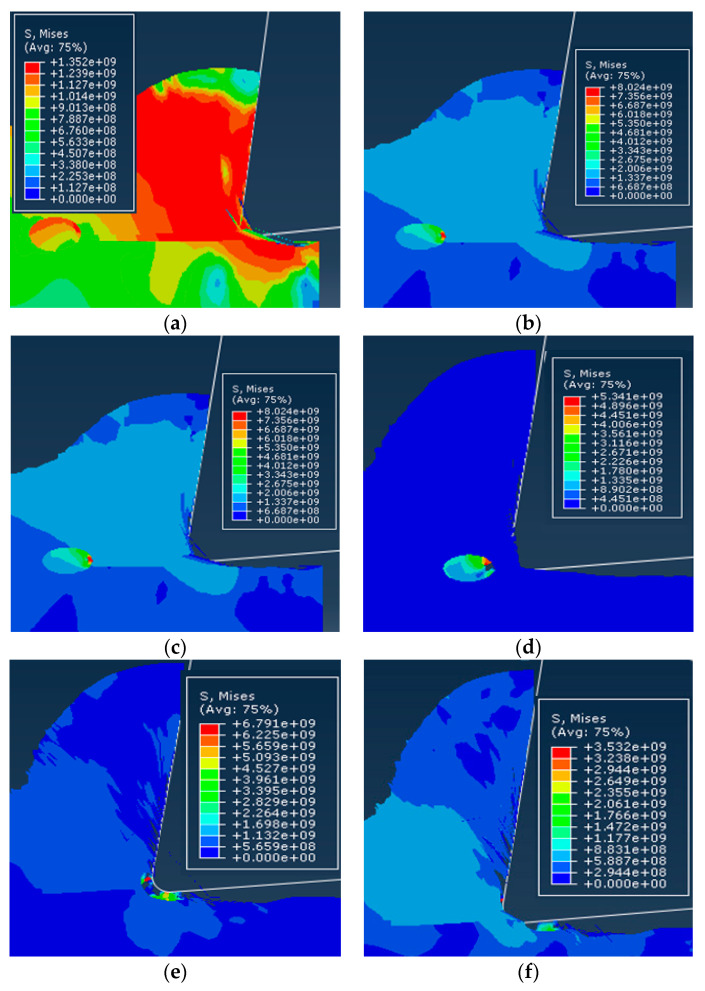
Simulation of the cutting process while the particles are positioned on the cutting path: (**a**) Stress concentration in the particle, (**b**) movement of the particle in position, (**c**) breakdown of particle, (**d**) particle is cut off, (**e**) particle removal is completed, (**f**) part of the particle is left on the cutting surface.

The simulated cutting process with the particles on the cutting path is shown in Figure 15, where the center of the particles and the center of the tool tip lie on the same horizontal line.

As the tool cut into the workpiece material along the cutting direction, near the particles, the stress concentration first occurred near the tip of the tool and the stress was greater than the surrounding matrix, as shown in Figure 15a. Due to the deformation and extrusion of the matrix, the force of the particles gradually increased. When the principal stress was greater than the tensile strength and reached the brittle fracture criterion, the SiC particles started to crack, and the cracks on the two sides with the greatest stress appeared successively. As the tool was further cut, the pushing action of the tool and the plastic deformation of the substrate caused the overall position of the particle to move forward and downward in the cutting direction, as shown in Figure 15b. When the tool was in contact with the particles, the force on the particles increased sharply, and the area of the broken particles increased, as shown in Figure 15c. The particles were then cut in the middle as a whole, as shown in Figure 15d. The part of the cut off particles became a part of the grains as the excised matrix rose along the rake face of the tool, and the other part directly separated from the matrix into chips; as shown in Figure 15e. When the tool completely cut through the particles, the particles were removed and the remaining of the particles were left on the cutting surface as shown in Figure 15f.

**Figure 16:**
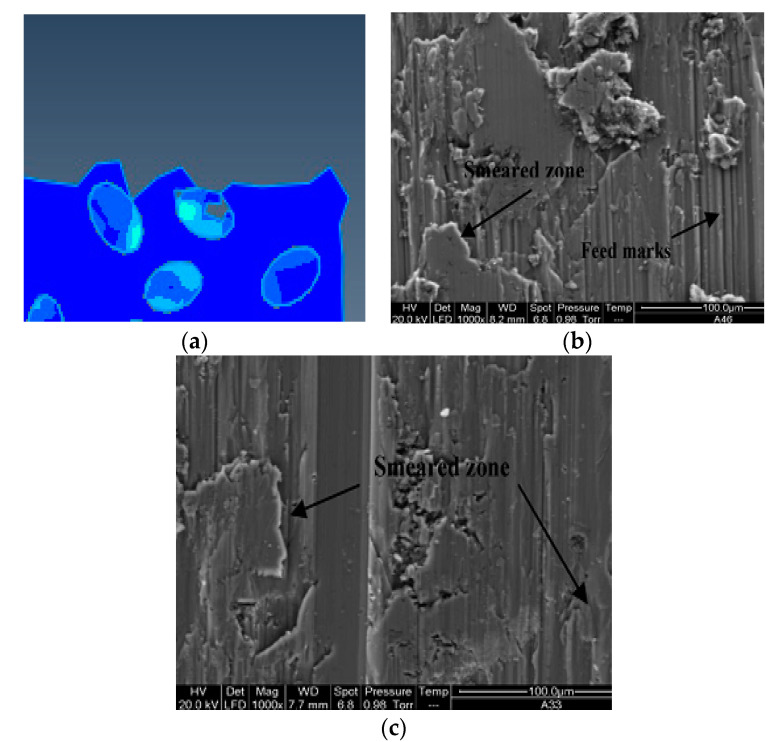
Micro cracks on the processed surface: (**a**) Simulation of surface topography cut through and feed marks, (**b**) cut through actual surface topography, (**c**) feed marks actual surface topography. (**b**,**c**) Adapted with permission from ref. [36] Copyright 2007, Elsevier.

**Figure 17:**
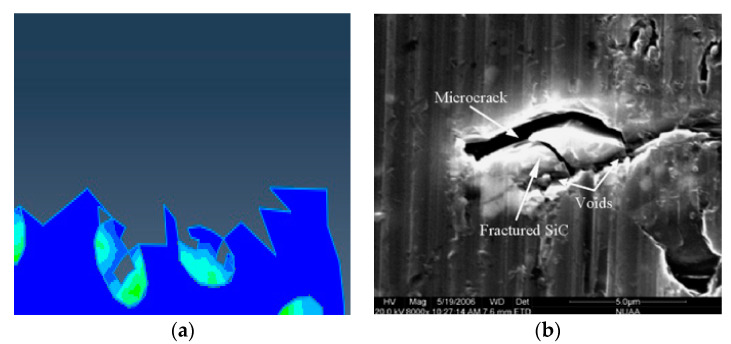
SiC particle cut morphology: (**a**) Simulation of particle cut off, (**b**) particle crushed and partial cut off morphology in actual processing. (**b**) Adapted with permission from ref. [37] Copyright 2008, Elsevier.

From the cutting process, it can be seen that the particles located on the cutting path were subjected to greater forces during the cutting process, and the failure of the particles was mainly manifested as brittle fracture of the particles. Due to the plastic deformation of the substrate and the movement of the particle position, unevenness of the cutting surface was caused and micro cracks appeared on the cutting surface, as shown in Figure 16. During the particle removal process, due to the fracture of the particles themselves and the plastic deformation of the matrix, gaps appeared between the particles and the surrounding matrix on the processed surface after the particles were removed, as shown in Figure 17.

In addition, when the cutter cuts particles in the actual processing, the particles may be directly removed as a whole because the particle size is larger or the cutting force is larger than the binding force between the particles and the substrate.

### 3.2. The Particles Were Located above the Cutting Path

**Figure 18:**
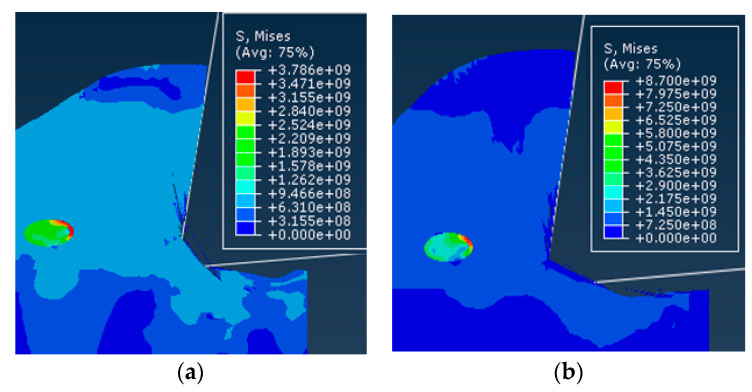

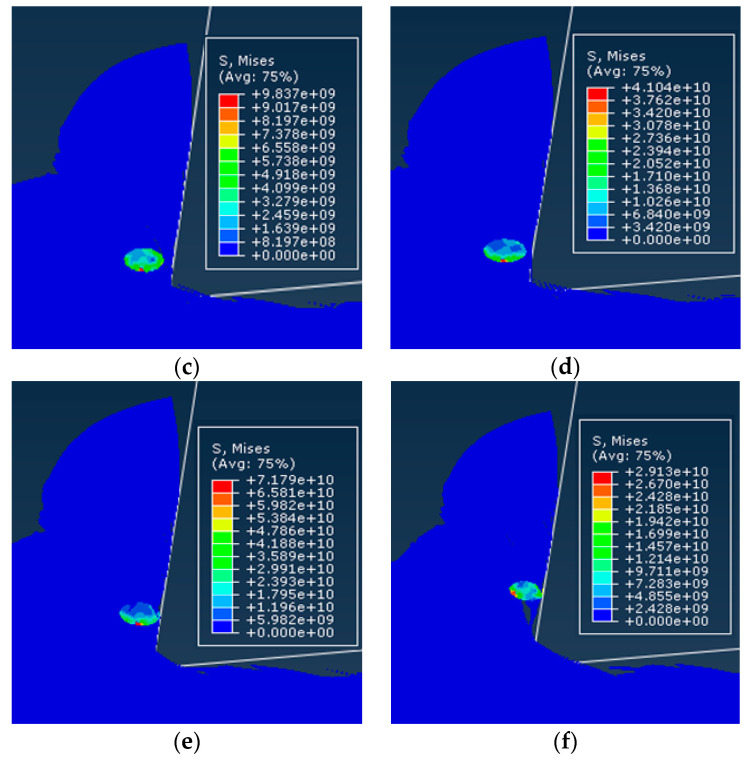
Simulation of the cutting process while the particles are positioned above the cutting path: (**a**) Stress concentration in the particle, (**b**) rising of particle along the rake face, (**c**) maximum forces under the particle, (**d**) particle begin to peel off from the matrix, (**e**) part of the matrix adheres to the particle, and (**f**) particle gradually break away from the chips.

The simulated cutting process where the particles were located above the cutting path is shown in Figure 18, where the center of the particles is located above the center of the tool nose. Similar to the simulation scenario in which the particles were facing the cutting path, as the tool advanced, the particles were first concentrated near the tip of the tool, as shown in Figure 18a, and as the substrate is distorted, the particles rose along with the chip along the rake face as shown in Figure 18b. Since the particles were located in the first denatured zone of the metal cutting zone, the plastic deformation and force of the chip were greatest here, and the area where the particles were subjected to the greatest force also became below the particles near the root of the chip, as shown in Figure 18c. As the tool was further cut in, the particles rose further along with the chips, and the particles began to peel off from the substrate near the rake face, as shown in Figure 18d. The particles were further exfoliated from the matrix and the matrix part attached to the particles was adhered to the particles, as shown in Figure 18e. The particles continued to rise along the rake face and began to come in contact with the rake face. Due to the friction between the particles and the tool, the degree of particle exfoliation increased, there was a tendency to detach from the grains, and the matrix fragments bonded to the particles, from the particles Separation, as shown in Figure 18f.

### 3.3. The Particles Were below the Cutting Path

**Figure 19:**
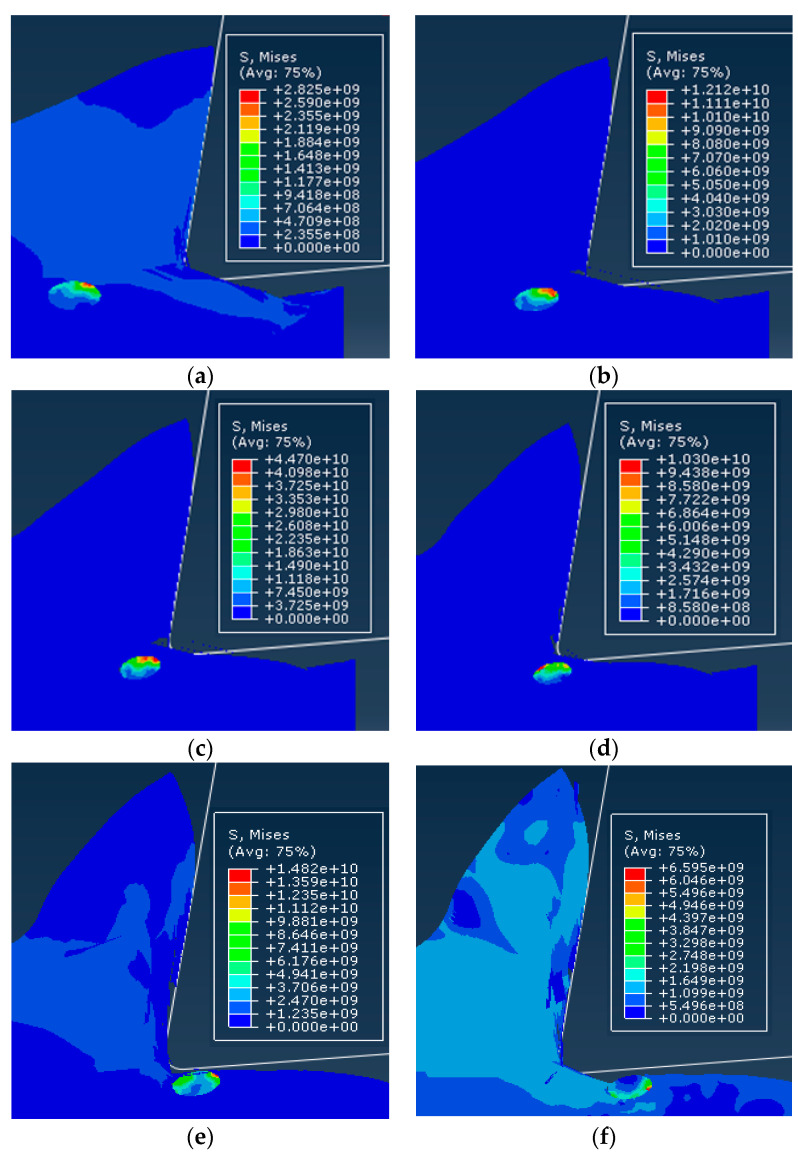
Simulation of the cutting process while the particles are below the cutting path: (**a**) Increased force above the particle, (**b**) movement of the particle, (**c**) partial separation of the particle, (**d**) broken of particle, (**e**) increased particle breakage areas, and (**f**) particle pressed into the matrix.

The simulated simulation cutting process where the particles were located below the cutting path is shown in Figure 19, where the center of the particles was located below the center of the tool nose and the majority of the particles were located below the cutting path. When the tool cut into the workpiece material gradually close to the particle due to the extrusion of the matrix above the particle, the force gradually increased, as shown in Figure 19a. With the formation of chips, the position of the particle near the root of the chip gradually moved forward and downward with the plastic deformation of the substrate, as shown in Figure 19b. The plastic deformation of the matrix around the particles increased, and the particles were peeled from the matrix, resulting in voids, as shown in Figure 19c. With further cutting of the tool, the force of the particle gradually increased and the particle near one side of the tool tip first began to break due to their principal stress exceeding the brittle fracture criterion, as shown in Figure 19d. As the cutters started to contact the particle the force of the particle increased sharply and the rupture area increased, as shown in Figure 19e. Due to the squeezing action of the tool flank, the particle was gradually pressed into the matrix and the part of the particle near the flank was removed, as shown in Figure 19f. The tool cut through the particle and continued to cut the matrix. As the force on the matrix around the particle decreased, the plastic deformation of the matrix as an elasto-plastic material decreases, the particle recovered with the deformation of the matrix, and the position also rebounded.

**Figure 20:**
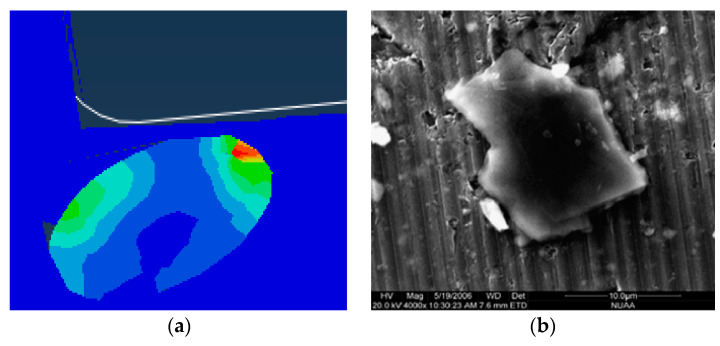
Particles pressed into the matrix: (**a**) Particle pressed into the substrate’s simulation topography, (**b**) particle pressed into the matrix topography during actual processing [37]. (**b**) Adapted with permission from ref. [37] Copyright 2018, Elsevier.

From the cutting process, it can be seen that the failure of the particles located below the cutting path was mainly the brittle fracture of the particles and the partial peeling of the particles from the matrix. Due to the pressing action of the tool, the particles were often pressed into the matrix, as shown in Figure 20, and after the cutting, a certain rebound occurred with the deformation of the matrix.

### 3.4. Effect of Interfacial Layer on SiC Particle Cutting

In order to study the influence of the interface layer on the cutting process and the cutting performance of materials, the simulation scenario of the particle on the cutting path was taken as an example. Based on this, the interface layer was introduced, and the modeling method of the interface layer was introduced.

**Figure 21:**
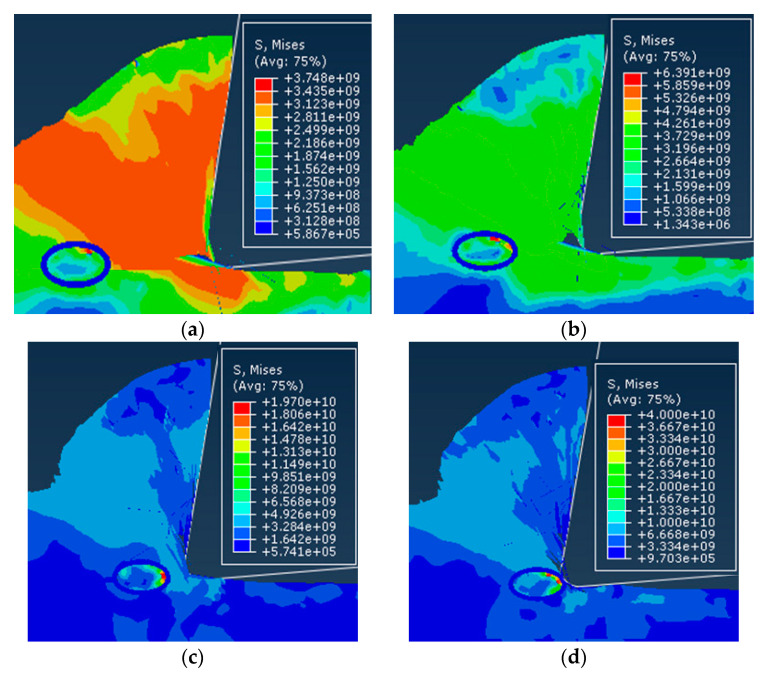

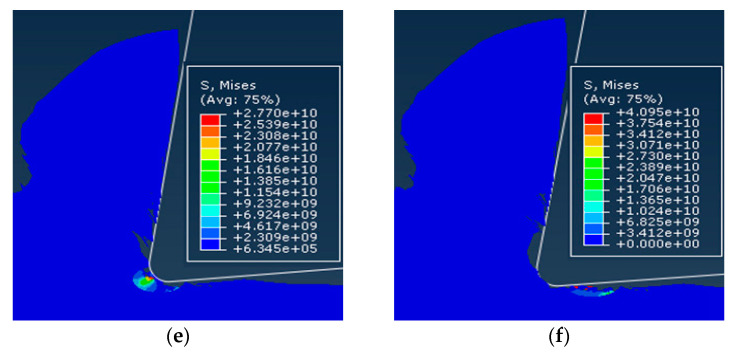
Model II simulation cutting process: (**a**) Stress concentration in the particle, (**b**) interfacial failure tool contact particle, (**c**) particle breakage, (**d**) increased particle breakage area, (**e**) complete particle removal, and (**f**) residual particle remain on the cutting surface.

Interface strength in the model $\sigma {MPa}_{max}$, Interfacial fracture energy $G^{c}=50{\;J/m}^{2}$, The actual thickness of the interface was 1.5 µm. Simulation cutting process is shown in Figure 21. For ease of explanation, the simulation model that has not been added to the interface layer is referred to as Model I, and the model that is added to the interface layer is referred to as Model II.

The cutting process of Model II was basically the same as Model I. The failure of SiC particles was mainly characterized by brittle fracture of particles, but there were also some significant differences. First of all, in the Model I, the stress concentration occurred first on the particle side near the tool. When the tool did not come into contact with the particles, the particles had already undergone partial rupture due to the stress reaching the brittle fracture criterion. In the Model II, although the stress on the particles was increasing during the approach of the tool, the brittle fracture criterion had not been reached until the tool broke, as shown in Figure 18a. Figure 18c shows the second fragmentation process of the particles in Model II was firstly the failure of the interface, the particle and the substrate were partially peeled off, and finally they were fractured under the cutting of the tool, as shown in Figure 21c,f. Finally, during the cutting process show the phenomenon that the movement of the particles due to the position of the tool was more pronounced in Model I than in Model II.

### 3.5. Effect of Interfacial Properties on Strengthening Properties of SiC Particles

For the interface layer parameters, for the sake of simplicity, according to the literature [38], it is assumed that the material properties of the substrate and the interface layer satisfy the following relationship:

(27) $$E_{i}=tE_{m}.$$

In the equation *i* and *m* respectively represent the interface layer and the substrate; *t*—the ratio of the elastic modulus of the interface layer to the substrate, called the interface stiffness coefficient. Elastic modulus can be used as an indicator to measure the degree of difficulty of elastic deformation of a material. The larger the value, the greater the stress required to cause a certain elastic deformation of the material, the greater the material stiffness, therefore, under certain stresses the smaller the elastic deformation.

At this time, *t* > 1 the elastic modulus of the interface layer is greater than the elastic modulus of the substrate, and the substrate may be plastically deformed earlier. In this scenario, the interface layer is called a rigid interface; otherwise, it is called a flexible interface. At the same time, the convention r denotes the interface layer thickness.

The strengthening of the particle-reinforced metal matrix composites is mainly due to the fact that the reinforcing particles have a high modulus of elasticity and the load is transferred to the particles. On this basis, through the post-processing of the finite element simulation results, the model stress changes under different simulation scenarios are quantitatively analyzed, and the influence of interface layer properties on the SiC particle strengthening performance is studied.

In the interface layer simulation study, the influence factors of interface layer thickness and interface stiffness coefficient are considered simultaneously, and different simulation examples are set up. The specific calculation example is shown in Table 9.

In order to analyze the stress distribution and its variation in the model, the average stress of the corresponding element in the model is first extracted, and then the following equation is used to post-process the finite element simulation results.

(28) $$\sigma_{c}=\frac{1}{S_{c}}\left[\overset{N_{c}}{\underset{k}{\sum }}\sigma_{ck}S_{ck}\right]$$

(29) $$\sigma_{m}=\frac{1}{S_{m}}\left[\overset{N_{m}}{\underset{k}{\sum }}\sigma_{mk}S_{mk}\right]$$

(30) $$\sigma_{p}=\frac{1}{S_{p}}\left[\overset{N_{p}}{\underset{k}{\sum }}\sigma_{pk}S_{pk}\right]$$

In the equation, *c*, *m*, and *p* respectively represent the composite material, matrix, and particle; [*σ_c_*] is the overall stress of the composite material; [*S_c_*] is the total element area of the composite material; [*S_ck_*] is the average stress of the kth element; and *N_c_* it is the total of the composite material and the total number of units.

In order to more directly analyze the influence of the interfacial property enhancement in the model, a proportional coefficient was introduced. *σ_c_*/*σ_m_* among them *σ_c_*, *σ_m_*. The overall stress of the composite material and the stress of the matrix phase are respectively indicated. The higher are the value, the less are the load that the matrix bears on the composite material, so that the SiC particles are subjected to a larger load and play a better reinforcing effect.

**Figure 22:**
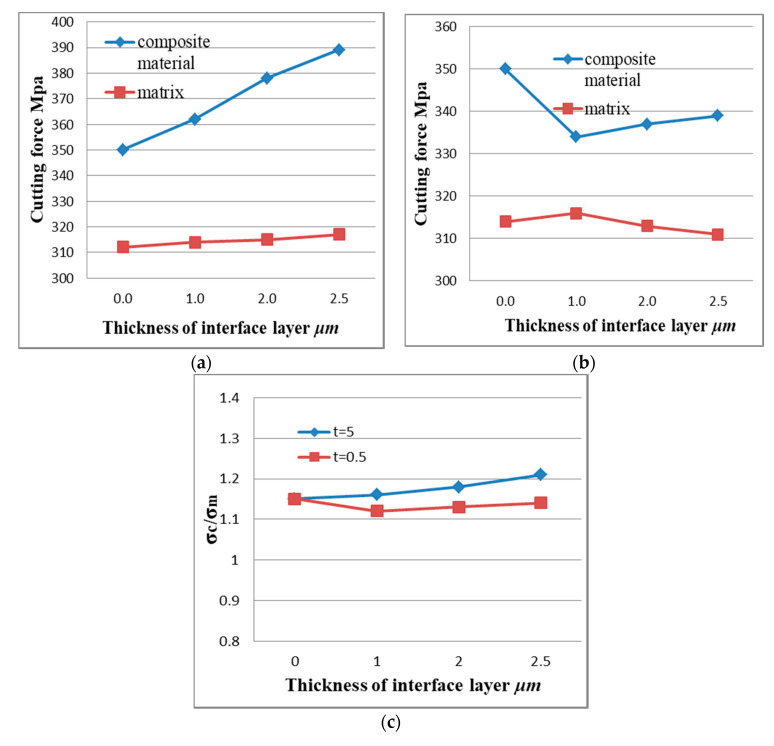
Effect of interface layer properties on composite stress: (**a**) Flexible interface (*t* = 0.5), (**b**) rigid interface (*t* = 5), and (**c**) proportional coefficient.

The stress of the matrix and the composite material in the different case models under the same conditions was calculated using the above equation; the results are shown in Figure 22.

For the flexible interface model, it can be seen from Figure 22a, that the stress difference of the substrate in the different interface layer thicknesses including the non-interface layer model was small. For the composite material as a whole, the stress in the interface layer was slightly less than that in the non-interface layer. Stress and the effect of interface layer thickness were small. This shows that when the interfacial layer between the particles and the matrix was a flexible interfacial layer, the strengthening effect of the reinforcing particles on the matrix material was relatively weak. The reason for the analysis may be mainly due to the fact that when the flexible interface model was deformed by force, the elastic modulus of the interface layer was smaller than that of the base material, so that the first plastic deformation occurs, and the large load was received and after breaking the load was reduced. The ability of the particles to enhance the particles will not be able to exert their strengthening effect. Therefore, the composite material of the flexible interface cannot obtain high strength.

For the rigid interface model, it can be seen from Figure 22b, that the stress range of the matrix under the rigid interface was still very small, but the overall stress of the composite material increases significantly with the increase of the thickness of the interface layer in the model. The reason for the analysis may be mainly due to the fact that the composite material undergoes plastic deformation rather than a rigid interface after stress. During the plastic deformation, the matrix transferred most of the load to the reinforcing particles through the rigid interface, thereby enhancing the particles which play its strengthening role. Therefore, the composite material of the rigid interface had a higher strength than the composite material. Scale factor from Figure 22c, *σ_c_*/*σ_m_*. From the fold line can be seen that with the appearance of the interface layer and the increase of the interface thickness, the enhancement effect was not obvious for the flexible interface composite material; the reinforcing effect of the rigid interface composite material was significant compared with the non-interface layer model, and the enhancement effect was significant, positive correlation with interface layer thickness.

## 4. Simulation of Cutting Process of SiCp/Al Composites

Based on the simulation study of SiC particle cutting, the cutting process of SiCp/Al composite material was simulated. The effects of cutting speed and cutting thickness on the surface morphology, chip shape, stress distribution, and cutting force were studied.

### 4.1. Effect of Cutting Speed

**Figure 23:**
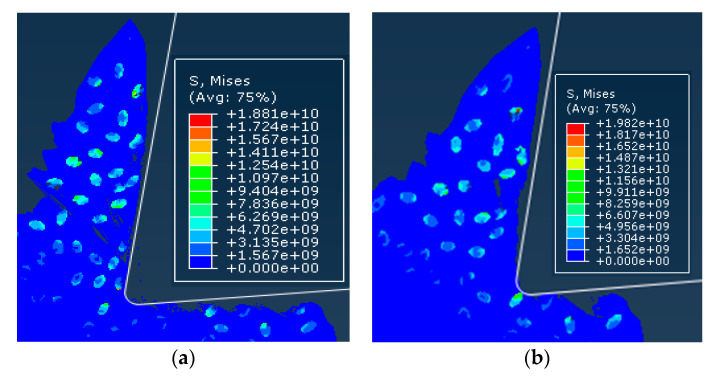
Comparison of stress distribution of different cutting speed examples: (**a**) *v_c_* = 100 m/min, (**b**) *v_c_* = 200 m/min.

In order to compare and analyze the effects of different cutting speeds *v_c_* on the cutting process, simulations were performed for cutting speed of *v_c_* = 100 m/min and cutting speed of *v_c_* =200 m/min. The simulation results were consistent for both models except for the cutting speed. Simulation results are shown in Figure 23.

Since the simulation conditions such as cutting depth and particle distribution were the same, the cutting speed had little effect on the failure condition of the particles and the cutting surface morphology. Because the particles were dragged by the tool on the cutting surface, the particles and the substrate were removed after the cutting tool was removed. The connected parts were easily peeled off to form burrs. The particles that were on the cutting path were broken and the particles located under the cutting path were pressed into the substrate by the cutter. As the cutting speed increases, the cutting surface quality was slightly better. From the chip morphology point of view, the chips showed a sharp saw-toothed shape, and the chips contained broken particles. With the further cutting, the chips appeared partial fracture. From the stress distribution point of view, the higher stress was also distributed at the tip of the particle and the position where the particle and the tool and the particle were in contact with the particle. When the stress exceeded the brittle fracture criterion of the particle occurred.

**Figure 24:**
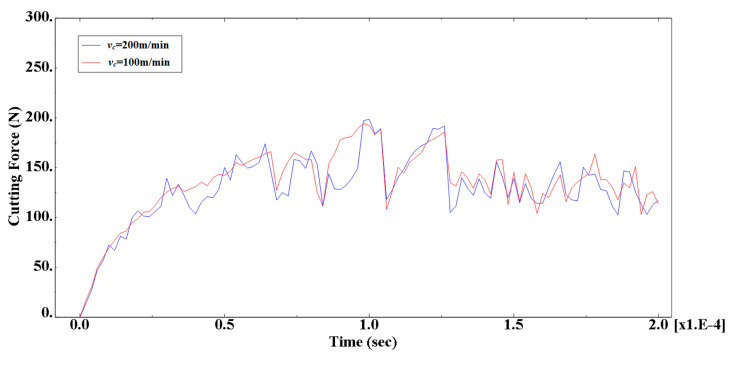
Comparison of cutting force for different cutting speeds.

In order to further analyze the effect of particle size on cutting force, simulation results of the main cutting force of the simulation model were extracted, as shown in Figure 24. From the cutting force change curve, it can be seen that the cutting force change trend was approximately the same, the cutting force value in the overall cutting process was generally the same, and there was a difference in the peak value at some positions. The reason for the analysis is that, in addition to the two groups of models, the cutting speed was consistent with other simulation conditions. In particular, the cutting thickness and the distribution of particles were the same. This determines that the distribution of the substrate and the particles on the cutting path of the cutting tool was the same, and the changing trend of the cutting force was approximately the same.

### 4.2. Effect of Cutting Thickness

**Figure 25:**
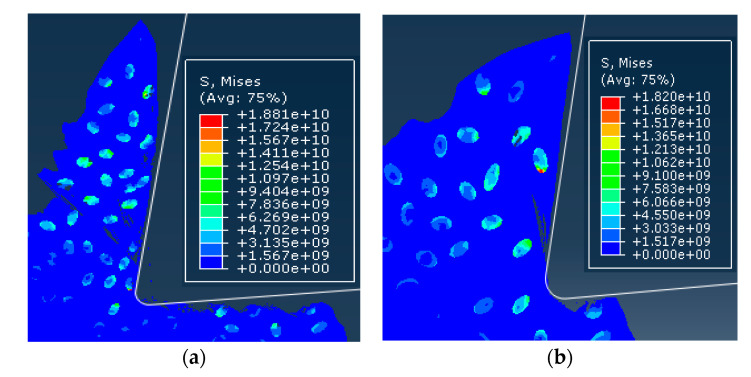
Comparison of stress distribution in different thickness calculations: (**a**) *a_c_*= 30 μm, (**b**) *a_c_* = 50 μm.

In order to compare the effects of different cutting thickness on the cutting process, simulations were performed for two cases with a cutting thickness of 30 μm and a cutting thickness of 50 μm. The simulation results were consistent with the simulation conditions except for the cutting thickness as shown in Figure 25.

From the cutting morphology, the increase in the thickness of the cut increases the size of the saw tooth and the number of particles contained in the cut chip. From the aspect of the processing surface morphology, it was basically the same as the standard example, still showing the appearance of burrs on the substrate surface and holes around the particles. From the stress distribution point of view, due to the increase in the number of particles in the cutting layer, the contact and squeezing effect between the particle and the particle was more obvious.

**Figure 26:**
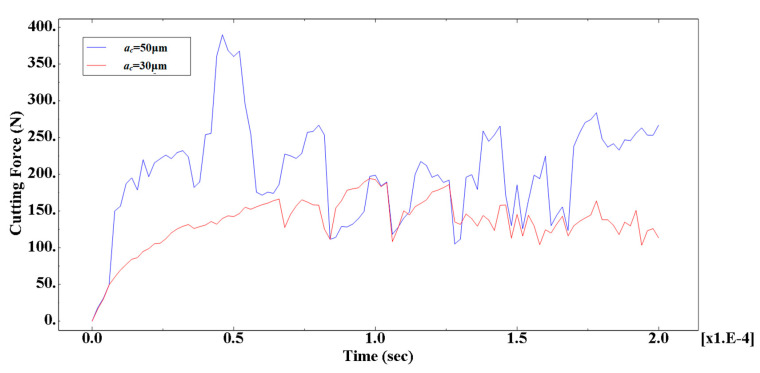
Comparison of cutting forces for different cutting thickness calculations.

In order to further analyze the effect of particle size on cutting force, simulation results of the main cutting force of the simulation model were extracted, as shown in Figure 26. From the cutting force change curve, it can be seen that the increase of the cutting thickness makes the value of the cutting force significantly increase during the cutting process, and the fluctuation of the cutting force is intensified. It can be seen that the cutting thickness was an important factor that affects the size of the cutting force.

The cutting thickness played a vital role as cutting thickness increased, the cutting force fluctuated more significantly, and as the cutting thickness increased, the fluctuation tended to increase gradually. This is because when the thickness increases, the cutting force was also increased, the blade–chip contact area was increased, and the number of particles in the blade-chip contact area increased, so that the rake face of the tool contacted more particles at the same time, which made the cutting force greater. At the same time, the increase of the thickness increased the cutting temperature and the plasticity of the material, which slowed down the fluctuation progression rate.

If the cutting force was constant during the cutting process, the workpiece material would generate more resistance to the tool in the cutting direction, resulting in an equal increase in the cutting force. However, the increase in cutting thickness would also reduce the deformation coefficient and reduce the cutting force. The combined effect of the positive and negative aspects would cause the cutting force to maintain a linear increase.

### 4.3. Effect of Cutting Parameters on Chip Geometry

In this study, the cutting force and chip formation behavior in machining of SiCp/Al containing 50% vol of SiC reinforced particles and 10 μm size was defined along with different machining parameters. In the cutting process, chip formation or chip removal mechanisms play a vital role to understand the machinability characters [39]. The patterns and types of chips in chip formation mechanisms in SiCp/Al material give clues to understand the effect of machining parameters surface quality as well as the influences on the cutting force generation patterns. Different cutting speed form different shapes and patterns of chips can be seen in figures that kind of phenomena also fluctuate the cutting force [40,41].

**Figure 27:**
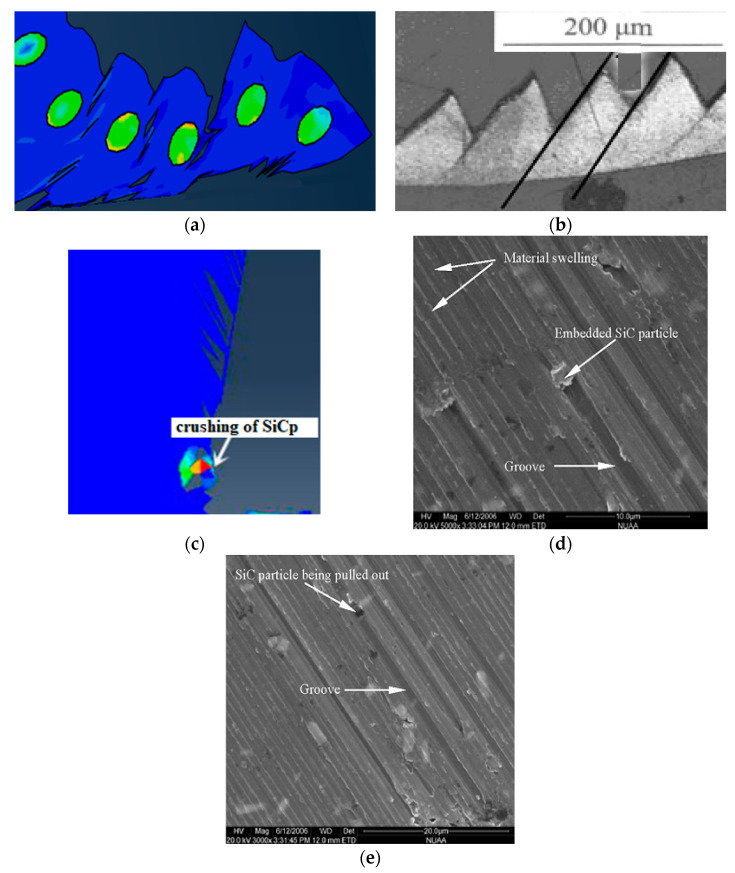
Change of chip geometry at different cutting parameters: (**a**) Simulation of chip morphology, (**b**) actual chip morphology (*v_c_* = 120 m/min), Copyright 2008, Elsevier [37], (**c**) simulation of chip morphology, (**d**) actual chip morphology, and (**e**) magnification at A, Copyright 2009, Elsevier [43].

It can be seen from the figure that the obtained chip geometry was mostly clockwork chips and C-shaped chips, and under different cutting conditions (cutting speed and feed rate), the radius of chip curl was significantly different. However, in the metal cutting process, chip curling was a very complicated process. At present, there is no accurate prediction model for measuring the roll radius of the chip. The researchers generally use the slip line field theory to explain the reason for this phenomenon [42]. The basic idea is that different flow rates at the two ends of the chip are the main reason for the chip to curl. It is caused by uneven strain in the secondary plastic deformation zone in cutting process. Therefore, the mechanism of chip curl deformation is caused by uneven strain or uneven strain rate in the second plastic deformation zone. From the chip geometries in Figure 27, it can be seen that the SiCp/Al composites materials studied in this work still maintain a certain plasticity mechanism. Therefore, this theory can be used to analyze the reasons for chip rolling mechanisms of the SiCp/Al composite materials.

As can be seen from Figure 27 that the cutting speed was gradually increase by the feed rate amount from 0.05 mm/r to 0.2 mm/r. During the cutting process, it is seen that the spring-shaped coils were formed frequently, but the chips were curled. The number of turns was gradually reduced, and the curl radius was gradually increased. The chips’ geometry phenomena change from spring-shaped coils to C-shaped chips mechanism, among them chip curl radius gradually increased. This is because when the feed rate is increased, the deformation coefficient is reduced, so that the chip roll-up radius is increased; the difference between the materials flow rate and the difference between the plastic deformation increases. At the same time, as the cutting speed increases, the cutting temperature also increases, and the tool–chip friction coefficient decreases and the chip friction coefficient plays a vital role in cutting force and chip geometry. The combination of two reasons and two factors causes the reduction of the deformation coefficient during the cutting process, which reduces the deformation coefficient, which leads to an increase of the chip rolling radius.

### 4.4. Effect of Particle Parameters on Chip Geometry

**Figure 28:**
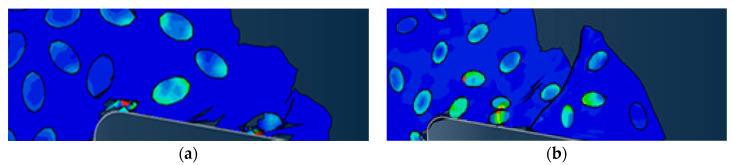
Factors affecting chip morphology: (**a**) Chip geometry changes with particle ratios 50%, (**b**) chip geometry changes with particle ratios 20%.

The different cutting parameters and materials structure can influence the chip formation. The different patterns of chip formation and evolution of chip shape can well reflect the mechanism of cutting force change. Figure 28 shows the evolution of the chip geometry of the SiCp/Al with different cutting granularities when cutting speed 100 m/min and cutting thickness of 30 μm and 50 μm respectively. It can be seen in Figure 28, as the particle size increased, the chip roll-up radius gradually decreased. This is because the SiCp/Al composite material has a very obvious particle size effect in chip formation process and its geometry. The larger the particle diameters in reinforced structure, the smaller the material’s yield stress and the easier the material to deform, resulting in a smaller chip rolling radius. This can be well explained that the changing trend of cutting force is due to the particle size. The larger the particle size, the easier to machine the SiCp/Al material, and the machining performance increases, and ultimately the smaller the cutting force required for the chip formation process.

Figure 28 shows the chip geometry of the cutting speed 100 m/min, cutting thickness of 30 μm and 50 μm respectively, particle diameter 10 μm, cutting two different volume fractions (20% and 50%) of SiCp/Al composite Shape change chart. It can be seen from the figure that as the particle volume fraction is larger, the chips change from the spring-shaped curling chips to the C-shaped chips. This is because the larger the volume fraction of the particles, the more the composite material tends to exhibit brittle properties, and it is easy to form chipping chips. This can well explain the reason for the high cutting force at high body weight ratio. The different flow velocities at both ends of the chip are the main cause of chip curling, as well as the uneven strain in the deformation zone. So, chip curling deformation is caused by non-uniform strain or non-uniform strain rate in the deformation zone. From the chip morphology in Figure 28 The plasticity mechanism of SiCp/Al composites is still maintained, so this theory can be used to analyze the cause of chip coiling of composites [44,45].

## 5. Chip Formation Mechanisms

### 5.1. Sawtooth Chip Formation Mechanism of SiCp/Al Composites

Studying the SiCp/Al composite chip morphology is convenient for us to understand the formation process of chip and the strengthening mechanism of SiC particles in the material. The law of influence plays an important role. Chip morphology can be mainly divided into band-shaped, sawtooth-shaped and nodular chips. Chip formation reported by Mabrouki et al. [45] Ozcatalbas et al. [46], Dabade and Joshi et al. [47,48], Opoz and Chen [39] that it is a combined crumbling/fracture/rupturing process with increasing shear plane angle; however, the partial shearing and tearing along shear plane reported in the work of Joshi et al. 1999 [48].

During its evolution, the formation of sawtooth shaped chips is a necessary stage for receiving continuous chips and nodular chips. Regarding the formation of sawtooth chips in the cutting process, there are two major theoretical systems to support it. First, the adiabatic shear theory scholars holding the view, which believes that the temperature rise caused by plastic deformation work in the shear zone makes the adiabatic shearing occurs in the cutting area, so that the shearing surface loses deformation resistance and material failure occurs which form the saw-tooth chips. The adiabatic shear theory can explain the phenomenon that the materials with low physical properties and general plastic materials are toothed at higher cutting speeds. The second is the periodic fracture theory [49]. There are three main ways for the accumulation and propagation of cracks in the cutting layer: (1) If the SiC particle size is small, the cracks bypass the SiC particles and continue to propagate in the matrix. (2) The contact strength of the interface with the reinforced particles is weaker; the dislocation plugging is easy to form at the interface between the SiC particles and the matrix, and the cracks follow the matrix. (3) The larger SiC particle size directly causes the reinforced particles to break through the crystals or break along the crystals. In the cutting process of SiCp/Al composite materials, under the action of the tool, micro-cracks in the shear zone quickly expand and aggregate to form the main crack, which causes the free surface of the chip to burst suddenly, thereby forming a saw-tooth chip.

### 5.2. Effect of Cutting Parameters on Chip Morphology

**Figure 29:**
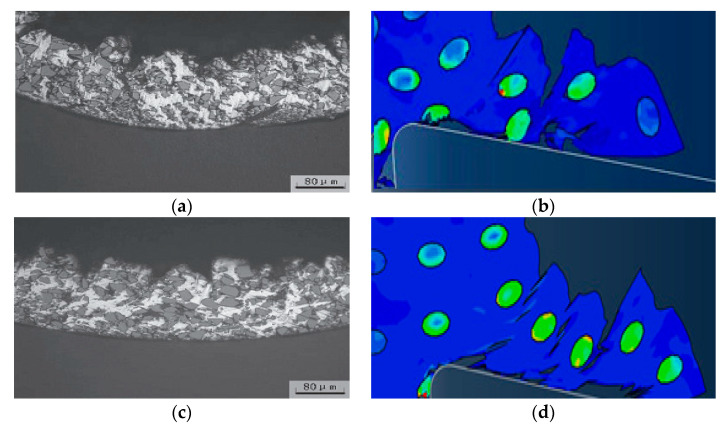

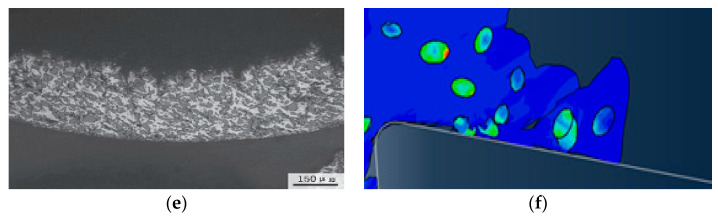
Shows the different cutting speeds compared to the effects of two feed rates on machining chip morphology and simulated chips morphology; chips obtained by machining the 2024Al/SiC-30 μm composite under various feed rates and cutting speeds and comparing SiCp/Al with simulated chips (**a**) shows chips at *v_c_* = 47 m/min and *f* = 0.1 mm/rev (**b**) shows simulated chips at *v_c_* = 40 m/min and *f* = 0.1 mm/rev, (**c**) shows chips at *v_c_* = 120 m/min and *f* = 0.1mm/rev, (**d**) simulated chips *v_c_* = 100 m/min and *f* = 0.1 mm/rev, (**e**) shows chips at *v_c_* = 120 m/min and *f* = 0.2 mm/rev, and (**f**) simulated chips at *v_c_* = 100 m/min and *f* = 0.2 mm/rev; (**a**,**c**,**e**) Adapted with permission from ref. [50] Copyright 2018, Elsevier.

Figure 29 shows the change of chip morphology of the SiCp/Al composite material with a feed rate of f = 0.1 mm/r and different cutting speeds. It can be seen from the figure that at low speed, the crack penetrates from the free surface of the chip to the bottom of the chip. Figure 29a,b is simulated chips obtained through Finite element analysis method. As the cutting speed increases, the depth of the shear crack extension phenomena possesses the reducing trend Figure 29c–f are simulated chips obtained through Finite element analysis method. The degree of chip sawing is slowed down, and there is a significant change in the chip morphology of the material, but the particle aggregation effect is more obviously seen. This is due to increase in cutting speed, which will cause the increments in the chip flow rate and increases the material strain rate, resulting in particles that are more likely to accumulate in the shear zone. The aggregation of such particles is more likely to cause the initiation of cracks, leading to an increase in the number of cracks result in the number of saw-tooth phenomena increases during machining process. On the other hand, an increase in cutting speed will increase the amount of material removal rate per unit time, which increases the cutting temperature and improve the plasticity of the SiCp/Al composite material. As a result, the fracture crack will only extend a certain distance towards the tool tip, and the extension will stop at a place where the compressive stress on the shear surface is large enough.

It can be seen that the different feed rates generate the strip-like chips, while with the increasing feed rate the chip formation behavior changes a bit with a decreasing number of coiling circles; however, the increment has seen in the radius of the coiling chips [47]. A higher amount of feed rate during the cutting process changes the spring-like coils in C-type coils and the curl radius increases. It is because the increment in feed rate reduces the coefficient of deformation, because of that chip coiling radius increases, at the same time increment in cutting speed increases the cutting zone temperature and improve the plasticity of SiCp/Al which provide easy machining process to form curling chips and reduce the cutting force [7,49].

**Figure 30:**
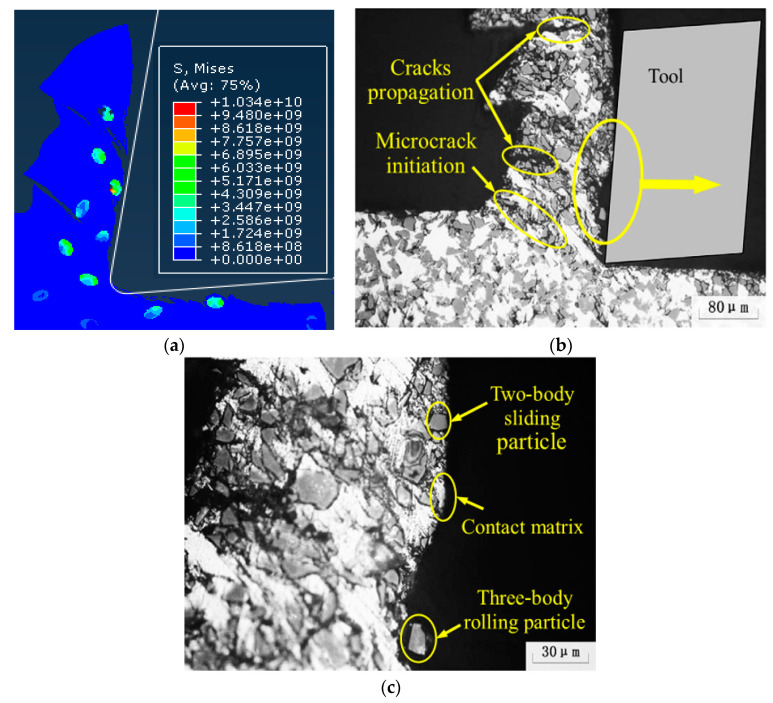
The chip morphology varies with particle size and cutting parameters: (**a**) The simulation of the chip formation of SiCp/Al. However, Chip formation in the machining of 2024Al/40%SiC-30 μm composites cutting speed of *v_c_* = 120 m/min and feed rate of *f* = 0.1 mm/rev, (**b**) a micrograph of the chip root, (**c**) an enlarged view of the three-phase friction at the tool–chip. (**b**,**c**) Adapted with permission from ref. [50] Copyright 2018, Elsevier.

Figure 30 shows the change in chip morphology of SiCp/Al 50% composites at a feed rate of *f*= 0.1mm/r and cutting speed *v_c_* = 100 m/min under different SiC particle sizes. As can be seen from Figure 30, with the same body fraction, as the SiC grain size increases, the composite chips gradually transform into band-shaped chips Figure 30c). At the same time, for materials with a larger SiC particle size, particles will be crushed and broken in the shear zone, and there will be a “spikey” phenomenon at the root of the chip. This is because the volume fraction is constant, the larger the SiC particle size, the less obvious the particle flow effect, and it is easy to be crushed and broken in the shear zone. At the same time, as the SiC particle size increases, the number of particles inside the composite material decreases, and the larger the contact area between the tool and the base material, the more easily the aluminum substrate will come into contact with the tool, resulting in a “spikey” phenomenon on the inner surface of the chip. At the same time, the larger the particle size, the greater the free path between the particle and the tool, which reduces the number of micro-cracks generated during cutting, improves the smoothness of the cutting process, and gradually changes the chip shape to band-shaped chips.

**Figure 31:**
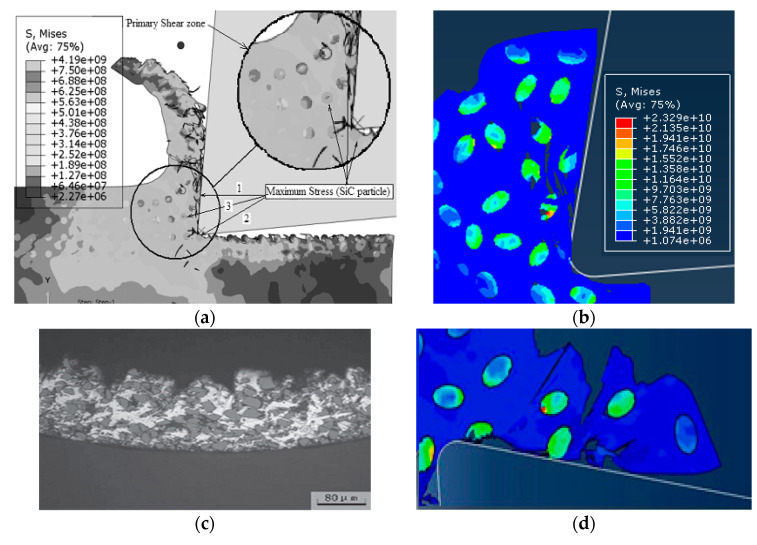
Comparison of chip morphology at different particle ratio, cutting speed and feed rate: (**a**) Particle ratio 20 μm *v_c_* = 80 m/min *f* = 0.24 mm/r. reproduced with permission from ref. [20] Copyright 2013, John Wiley and Sons, (**b**) simulated chip particle ratio 20 μm *v_c_* = 100 m/min *f* = 0.2 mm/r, (**c**) 30 μm *v_c_* = 120 m/min *f* = 0.1 mm/r. Adapted with permission from ref. [50] Copyright 2018, Elsevier, (**d**) simulated chip 20 μm *v_c_* = 100 m/min *f* = 0.1 mm/r.

Figure 31 shows the feed amount *f* = 0.1 mm/r, cutting speed *v_c_* = 120 m/min, *v_c_* = 100 m/min, *v_c_* = 80 m/min particle diameter 30 μm and 20 μm. Compare the effects of two volume fractions on chip morphology. It can be seen from the figure that at high volume fractions, the main crack inside the composite material penetrates the entire chip thickness and has a large degree of saw-tooth. This is due to the increase in the number of particles inside the material at high volume fractions and increased non-uniformity in the preparation of SiCp/Al composites, leading to increased defects such as micro-cavities and micro-cracks.

At the same time, the free path between the tool and the particles is reduced during the cutting process, resulting in an increase in micro-cracks. The combined effect of the two aspects leads to the accumulation of micro-cracks in the shear zone under the induction of shear stress, and the main cracks formed and penetrate the entire chip thickness.

The chip morphology of SiCp/Al material varies with the size of particles and reinforcement. It is found by Dabade and Joshi [47] that the particle size and reinforcement volume affect the chip formation as it increases the radius of curl on chips decreases. As the SiC particle size bigger it produces lower yield stress in the material, therefore the materials easily deformed. It is also seen that the cutting force fluctuates with SiC particle size in SiCp/Al composite materials, as the particle size is bigger in materials the lower cutting force required to remove the material and form the chips, and it produce ease in the cutting process and therefore reduces the machining force.

## 6. SiCp/Al Metal Matrix Composite Model Validation

### 6.1. Experimental Data Processing and Result Analysis

**Figure 32:**
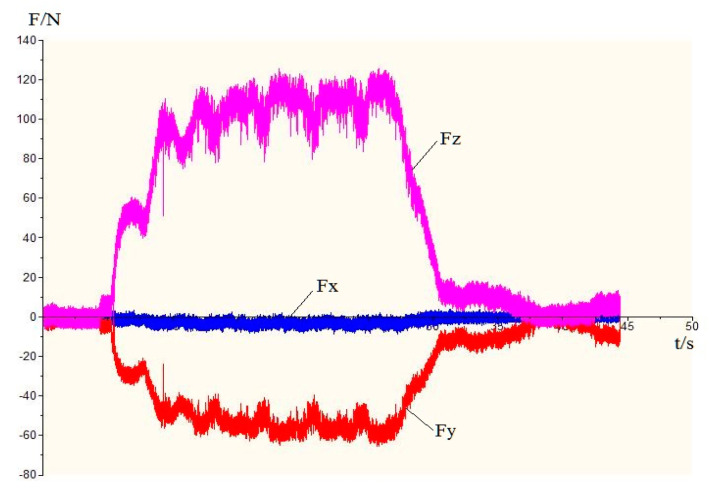
Curve of cutting force with time (*v_c_* = 80 mm/min, *f* = 0.05mm/r, *a_p_* = 2 mm).

This experiment uses DynoWare software to process the cutting force measurement results. The change trend of the cutting force in each group of experiments was roughly the same. Taking the third group of experiments as an example, the curve of the cutting force as a function of time is shown in Figure 32. In the figure, *F_x_* is the deep-cut resistance, *F_y_* is the feed resistance, and *F_z_* is the main cutting force. From the change in cutting force, it can be seen that due to the right-angle cutting experiment, the depth of cut is constant, and the depth-of-cut resistance *F_x_* is almost zero; the feed resistance and the main cutting force increase sharply at the beginning of the cutting and then tend to be stable. A certain range of values fluctuates and finally decreases to zero at the end of the cut. This is due to the sudden increase in cutting force when the tool encounters high hardness SiC particles during the cutting process, and the cutting force when the tool continues to cut the softer Al matrix after the SiC particles have been pressed into the matrix after cutting. Then it falls, which is consistent with the results of the simulation.

The signal analysis software DynoWare software was used to obtain the average value of the cutting force at each stage of the stable cutting operation. From the results of the cutting force, the value of the depth-of-cut resistance *F_x_* is very small and can be ignored. The main cutting force *F_z_* and the feed resistance *F_y_* all showed different degrees of increasing trend with the increase of the feed rate at different cutting speeds, and the main cutting force *F_z_* increased more obviously. From a numerical point of view, the main cutting force is greater than the feed resistance at different cutting speeds. The cutting force is changing with the increase of the cutting speed, which means that the feed rate is the main factor affecting the cutting force, and the cutting force increases with the increase of the feed rate.

### 6.2. Comparison between Simulated Cutting Force and Experimental Cutting Force

In order to compare and analyze the cutting force simulation and experimental results of SiCp/Al composites, the first, second, third, fourth, sixth, and fifteenth sets of right-angle cutting experiments were selected for simulation, in which the tool was re-modeled according to the experimental tool. The rake angle is 0° and the relief angle is 6°. The cutting parameters of each simulation are consistent with the corresponding experimental cutting parameters.

The simulation results under the same cutting conditions are compared with the experimental results and the deviation analysis is performed. The specific deviations between the cutting force simulation value and the cutting force experimental value are shown in Table 10.

**Figure 33:**
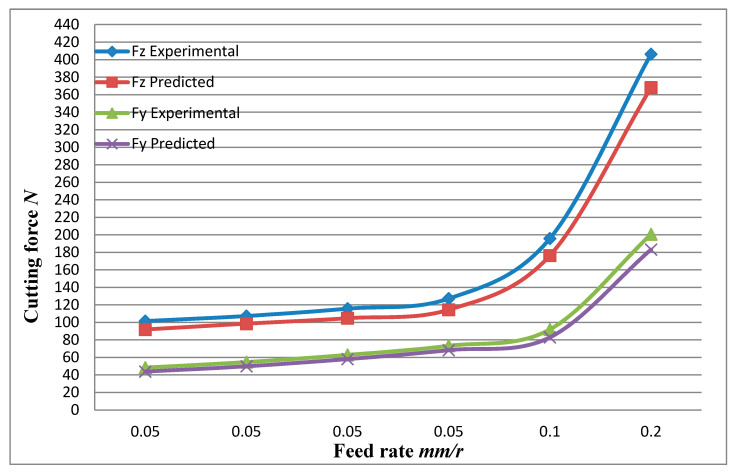
Comparisons of simulated cutting force and experimental values.

The data of the simulated cutting force and the experimental cutting force in Table 9 are plotted. As presented in a graph the cutting force varies with the feed amount and the cutting speed curve as shown in Figure 33. From the change curve of the cutting force in the figure, it can be seen that the changing trend of the simulated cutting force is consistent with the changing trend of the experimental cutting force. As the feed rate increases, both the main cutting force and the feed resistance increase significantly, and the main cutting force is greater than the feed resistance. With the increase of cutting speed, the main cutting force and feed resistance increase slightly, and the cutting speed has little effect on the cutting force. In addition, it can be seen from the analysis of the cutting force simulation value and the experimental value deviation that the cutting force simulation value and the experimental value are under 10%, which is basically within the acceptable range, and the cutting force experimental value is larger than the simulation. It is due to the fact that some values may vary because of the vibration of the machine tool during the cutting process and other factors.

## 7. Conclusions

In this paper, the machining processes of SiCp/Al metal matrix composite materials were studied. Through orthogonal cutting experiments and chip morphological observation, the influence of cutting speed, feed rate on the magnitude and fluctuation of cutting force, surface quality, and chip morphology during the cutting process were analyzed. The SiCp/Al composite cutting tool–chip interface friction was studied in-depth and a three-phase friction coefficient model of SiCp/Al composite cutting tool–chip contact was established. Based on the friction model, a two-dimensional random distribution model of SiCp/Al composite particles was developed by finite element method. The validity of the friction coefficient model was verified by comparing with experimental data. The specific conclusions are as follows:

- A finite element simulation model that is more consistent with the actual material composition and mechanical properties of SiCp/Al composites have been established, mainly reflecting the random distribution of particles in the matrix, the modeling of brittle cracking of particles, and the introduction of the interface layer cohesion model.
- The cutting state of the particles at different positions with respect to the tool cutting path was studied. The results show that the failure mode of the particles is mainly brittle fracture when it is facing the cutting path. The brittle fracture and the delamination of the particles from the matrix are mainly below the cutting path. The delamination of the particles and the matrix is mainly above the cutting path. The influence of interface layer on the cutting and strengthening of SiC particles was analyzed. The results show that the interface layer has a significant effect on the stress and strain transfer in the SiCp/Al metal matrix composite during the cutting process. For the flexible interface model, the enhancement effect does not change significantly with the increase of the interface thickness. For the rigid interface model, the enhancement effect is significantly better than without the interface layer model, and the enhancement effect increases as the thickness of the interface layer increases.
- The effects of different cutting parameters on the cutting process and cutting force of SiCp/Al metal matrix composite was investigated. The results show that the cutting force increases with the increase of cutting thickness, but the influence of cutting speed is not obvious. On the cutting surface, there are defects such as holes left by the crushing of the particles, burrs, and gaps formed by tearing of the matrix, and protrusions formed by particles pressed into the matrix and partially exposed to the outside.
- As the feed rate gradually increases, the chip geometry changes from spring-shaped coils to C-type chips, the number of curls gradually decreases, and the radius of the chip curls gradually increases. All three cutting speeds are easy to generate spring-shaped coils, and as the cutting speed increases, the chip rolling radius increases. As the particle size increases, the more the number of chip coils, the smaller the rolling radius.
- A single factor orthogonal cutting experiment was designed. From the experimental results, the feed rate is the main influencing factor of cutting force. Both the main cutting force and the feed resistance simulation value increase with the increase of the feed amount, which is consistent with the experimental results. In addition, the deviation of the cutting force simulation value from the experimental value is less than 10%, which verifies the accuracy of the model.

## Figures and Tables

**Table 1:** Aluminum matrix material parameters.

	Young’s Modulus *E* (GPa)	Poisson’s Ratio *ν*	Thermal Expansion Coefficient (K^−1^)	Density (Kg·m^−3^)	Thermal Conductivity κ (W·m^−1^·K^−1^)	Specific Heat Capacity *c* (J·Kg^−1^·K^−1^)
Aluminum Substrate	68.9	0.33	2.18 × 10^−5^	2.70 × 10^3^	193	900

**Table 2:** Johnson–Cook constitutive model parameters of aluminum matrix.

Parameters	A (MPa)	B (MPa)	C	n	m	Tmelt (K)	Troom (K)
Value	176.45	63.99	0.0036	0.7	0.31	923	293

**Table 3:** SiC particle material parameters.

	Young’s Modulus *E* (GPa)	Poisson’s Ratio *ν*	Thermal Expansion Coefficient (K^−1^)	Density (Kg·m^−3^)	Thermal Conductivity κ (W·m^−1^·K^−1^)	Specific Heat Capacity *c* (J·Kg^−1^·K^−1^)
SiC Particles	485	0.2	4.90 × 10^−9^	3.20 × 10^3^	81	427

**Table 4:** SiC particle brittle cracking material model parameters.

$\sigma_{tu}^{I}\;(\mathrm{MPa})$	$u_{n0}\;(J/m^{2})$	$u_{n0}\;(m)$
1500	30	4 × 10^−8^

**Table 5:** Material parameters matrix.

PROPS1	PROPS2	PROPS3	PROPS4	PROPS5	PROPS6
$\sigma_{n}^{max}$	$\delta_{n}$	$\sigma_{s}^{max}$	$\delta_{s}$	$T_{0}$	*E*
2.24 × 10^8^ Pa	1 × 10^−6^ m	5.8 × 10^7^ Pa	1 × 10^−6^ m	1 × 10^−3^ mm	3 × 10^5^ MPa

**Table 6:** State variable matrix.

SDV1	SDV2	SDV3	SDV4	SDV5	SDV6	SDV7	SDV8	SDV9	SDV10	SDV11
$\varepsilon_{22}$	$\varepsilon_{11}$	$\varepsilon_{33}$	$\varepsilon_{12}$	${∆}_{n}$	${∆}_{s}$	$\Phi_{n}^{0}$	$∆\Phi_{n}$	$\Phi_{s}^{0}$	$∆\Phi_{s}$	Fra

**Table 7:** Johnson–Cook failure model parameters for matrix materials.

*d*_1_	*d*_2_	*d*_3_	*d*_4_	*d*_5_	*u* ^−pl^
0.13	0.13	−1.5	0.011	0	4 × 10^−6^

**Table 8:** Single factor orthogonal cutting experimental parameters.

Experiment Number	Cutting Speed *v_c_* (m/min)	Feed Rate *f* (mm/r)	Depth of Cut *a_p_* (mm)
1	40	0.05	2
2	60	0.05	2
3	80	0.05	2
4	100	0.05	2
5	40	0.10	2
6	60	0.10	2
7	80	0.10	2
8	100	0.10	2
9	40	0.15	2
10	60	0.15	2
11	80	0.15	2
12	100	0.15	2
13	40	0.20	2
14	60	0.20	2
15	80	0.20	2
16	100	0.20	2

**Table 9:** Interface layer simulation example solution.

Examples	Interface Layer Thickness *r* (μm)	Interface Stiffness Coefficient *t*
1	1	0.5
2	1	5
3	2	0.5
4	2	5
5	2.5	0.5
6	2.5	5

**Table 10:** Cutting force simulation and experimental value Error % prediction table.

Number of Experiment	Cutting Speed (m/min)	Feed Rate (mm/r)	Main Cutting Force *F_z_*)			Axial Thrust Force (*F_y_*)		
			Experimental Value (N)	Predicted Value (N)	Error (%)	Experimental Value (N)	Predicted Value (N)	Error (%)
1	40	0.05	101.5	91.9	9.16%	48.4	43.8	9.5%
2	60	0.05	107.3	98.6	8.13%	54.7	49.9	8.77%
3	80	0.05	115.7	104.8	9.07%	62.9	58.1	7.6%
4	100	0.05	127.2	114.5	9.89%	73.3	68.3	6%
6	80	0.10	195.8	176.4	9.8%	91.98	82.9	9.87%
15	80	0.20	406.3	368.1	9.76%	200.6	183.2	8.67%
